# Influence of acceptors on the optical nonlinearity of 5*H*-4-oxa-1,6,9-trithia-cyclopenta[*b*]-as-indacene-based chromophores with a push–pull assembly: a DFT approach†

**DOI:** 10.1039/d3ra06673h

**Published:** 2024-01-03

**Authors:** Muhammad Khalid, Shahzad Murtaza, Khansa Gull, Saba Abid, Muhammad Imran, Ataualpa A. C. Braga

**Affiliations:** a Institute of Chemistry, Khwaja Fareed University of Engineering & Information Technology Rahim Yar Khan 64200 Pakistan muhammad.khalid@kfueit.edu.pk khalid@iq.usp.br; b Centre for Theoretical and Computational Research, Khwaja Fareed University of Engineering & Information Technology Rahim Yar Khan 64200 Pakistan; c Department of Chemistry, Faculty of Science, King Khalid University P. O. Box 9004 Abha 61413 Saudi Arabia; d Departamento de Química Fundamental, Instituto de Química, Universidade de São Paulo Av. Prof. Lineu Prestes, 748 São Paulo 05508-000 Brazil

## Abstract

Herein, a series of compounds (TPD1–TPD6) having a D–π–A architecture was quantum chemically designed *via* the structural modulation of TPR. Quantum chemical calculations were employed to gain a comprehensive insight into the structural and optoelectronic properties of the designed molecules at the M06/6-311G(d,p) level. Interestingly, all the designed chromophores displayed narrow energy gaps (2.123–1.788 eV) and wider absorption spectra (*λ*_max_ = 833.619–719.709 nm) with a bathochromic shift in comparison to the reference compound (*λ*_max_ = 749.602 nm and *E*_gap_ = 3.177 eV). Further, *E*_gap_ values were utilized to evaluate global reactivity parameters (GRPs), which indicate that all the chromophores expressed higher softness (*σ* = 0.134–0.559 eV^−1^) and lower hardness (*η* = 4.155–4.543 eV) values than the reference chromophore. Efficient charge transfer from donors towards acceptors was noted through FMOs, which was also supported by DOS and TDM analyses. Overall, the TPD3 derivative exhibited a remarkable reduction in the HOMO–LUMO band gap (1.788 eV) with a red shift as *λ*_max_ = 833.619 nm. Furthermore, it exhibited prominent linear and non-linear characteristics such as *μ*_total_ = 24.1731 D, 〈*α*〉 = 2.89 × 10^−22^ esu, and *β*_total_ = 7.24 × 10^−27^ esu, among all derivatives. The above findings revealed that significant non-linear optical materials could be achieved through structural tailoring with studied efficient acceptors.

## Abstract 2

Herein, a series of compounds (TPD1–TPD6) having a D–π–A architecture was quantum chemically designed *via* the structural modulation of TPR.

## Graphical abstract

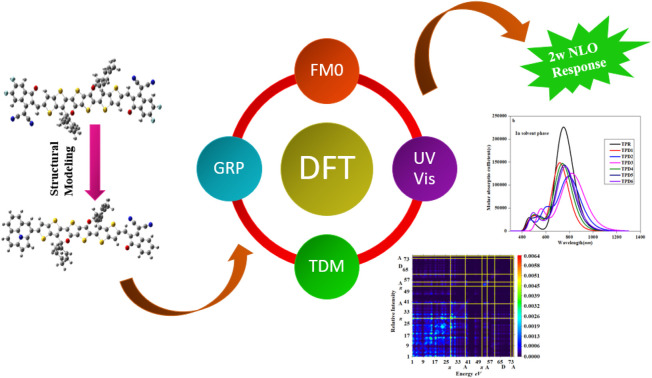

## Introduction

Nonlinear optics (NLO) is an interesting field in the area of modern optics, which is comparable to laser physics and focuses on studying various nonlinear phenomena that arise from the interaction between matter and lasers.^1,2^ Applications such as fiber optics, telecommunications and information management have greatly benefited from the utilization of NLO materials.^3,4^ Both organic and inorganic high-performance optoelectronic materials have revolutionized the area of research and technology.^5,6^ Moreover, the organic compounds with optoelectronic properties possess several advantages over inorganic ones, including fast response times,^7,8^ molecular flexibility, excellent processing capabilities, and rapid rates of polarization.^9,10^ Moreover, these organic chromophores exhibit wide band spectra and quick responses, which make them attractive for NLO applications.^11,12^ Their first hyperpolarizability is found to be associated with intramolecular charge transfer (ICT), which occurs from an electron-donating group (D) to an electron-accepting group (A) *via* π-conjugated linkers or spacers.^13^ They also possess second- and third-order NLO characteristics, which are particularly interesting owing to their potential applications in optical data processing and communications.^14^ NLO chromophores exhibit significant utility across diverse scientific domains, including nuclear research, medicine, chemical dynamics, solid-state physics, materials science, and biophysics.^15,16^ These compounds have linear and non-linear optical properties. The D–π–A star-shaped triazine derivatives have various applications, notably in optical computing, optical communication, and dynamic image processing.^17,18^ Consequently, researchers have increasingly shifted their focus towards organic compounds with optoelectronic properties in the past fifteen years, considering their ease of synthesis and low dielectric constants.^19–21^ The primary source of NLO characteristics in such compounds is the strong intramolecular charge transfer, which involves the transfer of electron clouds from the donor to acceptor segments *via* π-linkers. Structural modifications can be employed to achieve desirable NLO properties in organic materials, such as high susceptibilities, optical clarity, thermal stability, and solubility.^22^ The literature includes data on such structurally-modified frameworks, including donor–acceptor, donor–π–acceptor, donor–π–acceptor–π–donor, donor–π–π–acceptor, and donor–acceptor–π–acceptor.^23–26^

Non-fullerene acceptors (NFAs), compared to other π-conjugated frameworks, exhibited relatively high NLO outputs, which make them promising candidates in this field. The NFA-based compounds exhibiting NLO characteristics displayed tunable energy gaps as well as excellent stability; therefore, their optoelectronic properties can be tailored sufficiently to obtain desirable results.^27^ Considering the above-mentioned facts, we have selected a NFA compound (COi8DFIC) from the literature and proposed its data analysis. The compound's synthesis is reported by Zuo Xiao and co-workers.^28^ To the best of our knowledge, there is no currently available information regarding its nonlinear optical (NLO) properties. The compound (COi8DFIC) possesses a core unit with oxygen atoms acting as bridges, which demonstrate electron-donating characteristics. First, the oxygen atoms substantially enhance the electron-donating ability of core units *via* the conjugation effect. Second, they contribute to the expansion of the molecular backbone, leading to enhanced planarity that promotes favorable π–π stacking interactions imparting improved charge mobility within the molecule.^29^ Consequently, the presence of bridging oxygen atoms in its core unit contributes to an enhanced NLO response in COi8DFIC. Further, some innovative D–π–A-based derivatives are fabricated from the A–π–A parent compound (COi8DFIC), which is done using some efficient acceptor species from the literature. The resulting compounds possess efficient push–pull mechanism with a π–conjugation to achieve high NLO outputs. For this purpose, the Minnesota functional approach is utilized (density functional theory, termed DFT) due to the fact that their statistics match best with the experimental data of compounds. Moreover, they have significantly advanced the prediction accuracy of charge transport and electro-optical properties.^30,31^ DFT has long been considered the simplest and cost-effective approach for predicting NLO properties of organic materials.^32,33^ Therefore, DFT/TDDFT methods have gained much interest in the past several decades.^34–36^ These NLO-based findings could provide some insights for the development of novel organic entities with the D–π–A architecture, which are considered to be fullerene-free. The present research could also help researchers and experimentalists to develop potential NLO materials that exceed present expectations.

## Computational procedure

For all computational investigations, the M06 functional^37^ and 6-311G(d,p) basis sets^38^ were employed to optimize the molecular geometries without imposing any restrictions on the symmetry. The Gaussian 09 program^39^ provided by the lab of Dr Ataulpa Albert Carmo Braga was utilized for this purpose. The optimized structure as well as the input files of investigated compounds were generated using the GaussView 6.0 (ref. 40) software. The electronic properties included the energies of the highest occupied molecular orbitals (HOMOs) and the lowest unoccupied molecular orbitals (LUMOs) along with their differences determined in the frontier molecular orbital (FMO) analysis by utilizing the Avogadro software.^41^ Moreover, the absorption spectra (UV-Vis) and natural bond orbital (NBO) analysis of TPR and TPD1–TPD6 were also determined using software such as GaussSum,^42^ Origin 8.5 (ref. 43) and NBO program 3.1.^44^ Similarly, the transition density matrix (TDM) analysis and binding energies of compounds were calculated using the Multiwfn 3.7 (ref. 45) software. The same software was utilized in calculating the NLO parameters, which included dipole moment (*μ*),^46^ linear polarizability 〈*α*〉,^47^ first hyperpolarizability (*β*_total_)^48^ and second hyperpolarizability (*γ*_total_).^48^

Dipole moment^49^ was calculated using the following equation:

1 *μ* = (*μ*_*x*_^2^ + *μ*_*y*_^2^ + *μ*_*z*_^2^)^1/2^

Similarly, the 〈*α*〉,^50^*β*_total_ (ref. 51) and *γ*_total_ (ref. 52) were determined using the following equations:

2 〈*α*〉 = (*a*_*xx*_ + *a*_*yy*_ + *a*_*zz*_)^1/3^

3 *β*_total_ = (*β*_*x*_^2^ + *β*_*y*_^2^ + *β*_*z*_^2^)^1/2^

where *β*_*x*_ = *β*_*xxx*_ + *β*_*xyy*_ + *β*_*xzz*_, *β*_*y*_ = *β*_*yyy*_ + *β*_*xxy*_ + *β*_*yzz*_, *β*_*z*_ = *β*_*zzz*_ + *β*_*xxz*_ + *β*_*yyz*_4
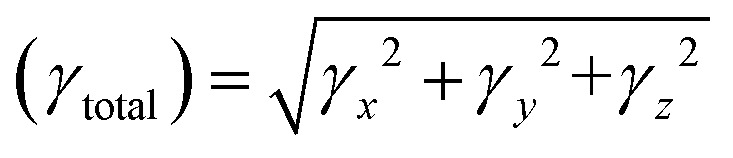
where 
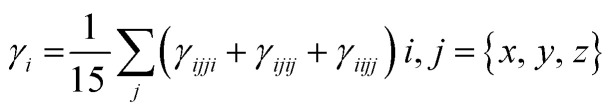


Other software were PyMolyze^53^ and Chemcraft 1.6 (ref. 54) to analyze the results from output files.

## Results and discussion

**Fig. 1:**
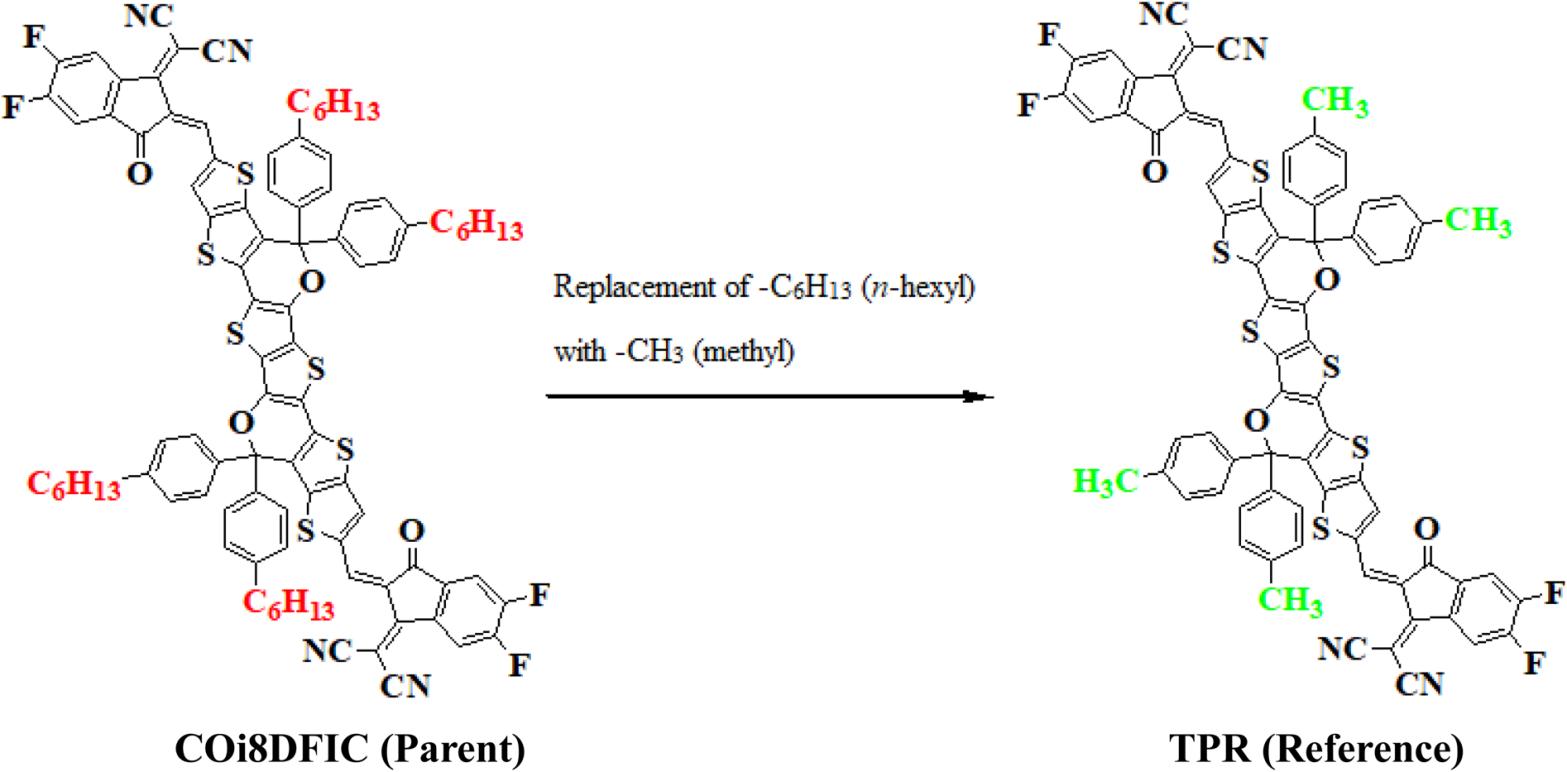
Structural fabrication of the parent compound (COi8DFIC).

**Scheme 1:**
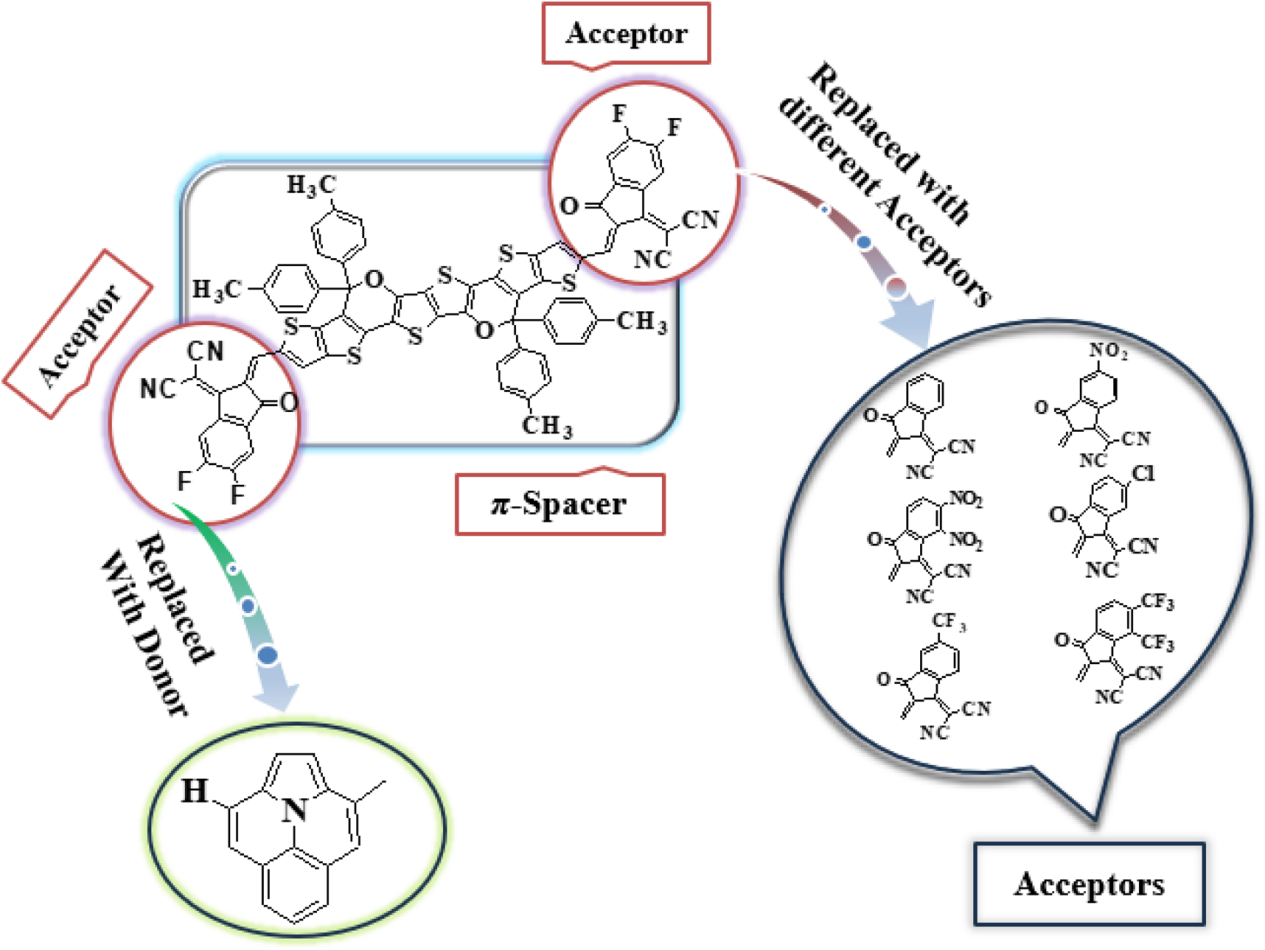
Graphical representation of TPR and TPD1–TPD6 derivatives.

**Fig. 2:**
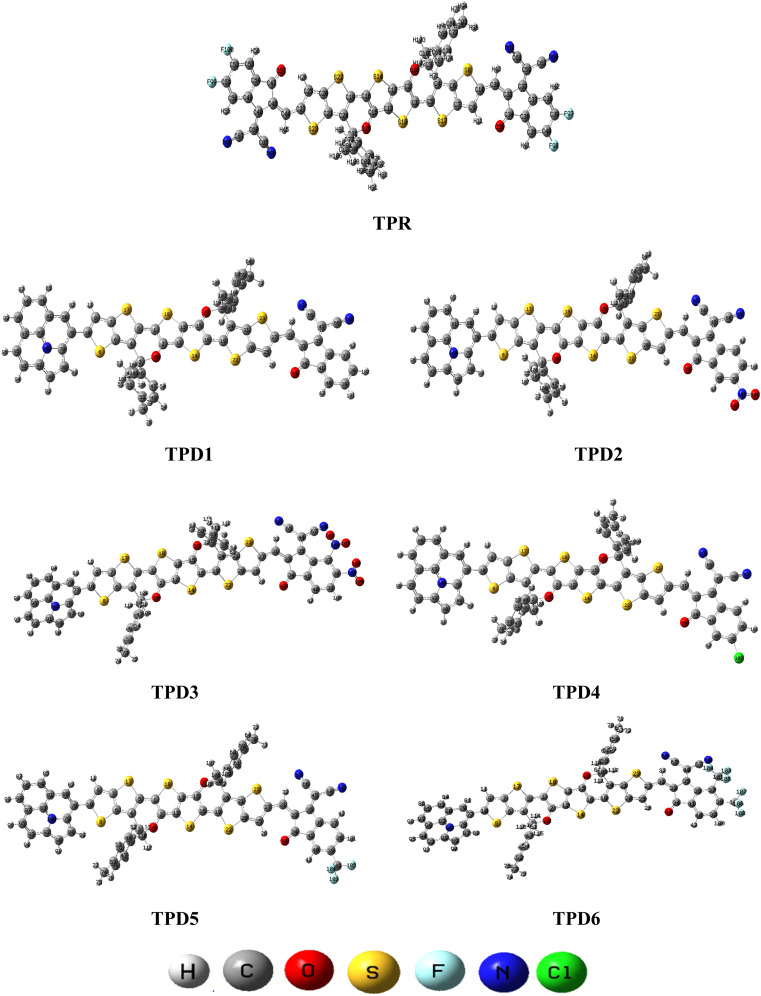
Optimized structures of the reference (TPR) and designed compounds (TPD1–TPD6).

In this study, we conducted a comprehensive investigation of NLO properties of a series of designed compounds to explore their potential for optoelectronic applications. Specifically, we focused on the NLO response of six different compounds derived from TPR, namely, TPD1–TPD6. The TPR compound is derived from the parent compound (COi8DFIC) by modifying the bulky *n*-hexyl groups with methyl groups, as shown in Fig. 1. Further, the A–π–A structure of TPR is modified into a D–π–A configuration by incorporating an efficient donor group^55^ named 9b-aza-cyclopenta[*cd*]phenalene reported in the literature. The resulting derivative is termed TPD1, while in the rest of derivatives (TPD2–TPD6), unique acceptor^56^ species are used, which replaced the acceptor group of TPD1, while the donor species is retained. Scheme 1 represents the structural modification of the parent compound in order to design unique NFA-based derivatives. The chemical structures of all the entitled molecules are shown in Fig. S1,† while their optimized geometrical structures are displayed in Fig. 2. Within Tables S1–S7,† the detailed presentation of Cartesian coordinates for the respective data sets is illustrated. By adopting a D–π–A configuration, our aim is to enhance the optoelectronic properties and NLO characteristics of the designed compounds. The main objective of our study is to assess the NLO performance of the designed compounds and gain an insight into their potential for applications in optoelectronics. Moreover, by systematically varying the acceptor groups, we also explored the influence of different electron acceptor units on the NLO properties of compounds. To achieve this, we performed their quantum chemical investigation by using DFT/TDDFT, *i.e.*, M06/6-311G(d,p) functional and analyzed the (i) energy gaps (*E*_g_), (ii) UV–Vis absorption (*λ*_max_), (iii) stabilization energy (*E*^(2)^), (iv) global reactivity depicters such as ionization potential (IP), electron affinity (EA), electronegativity (*X*), global softness (*σ*), hardness (*η*) and electrophilicity index (*ω*), (v) binding energy (*E*_b_), (vi) HOMO–LUMO contributions (DOS) and (vii) NLO properties *i.e.*, 〈*α*〉, *μ*_total_, *β*_total_ and *γ*_total_. The present research would possibly allow the experimentalists to synthesize these compounds in the future.

### Frontier molecular orbitals (FMOs)

FMO analysis is an important tool to elucidate the chemical stability, optical as well as electronic properties of molecules.^57^ The charge transmission of molecules is largely influenced by the redistribution of FMOs, *i.e.*, HOMOs and LUMOs. According to band theory, HOMO refers to the valence band and LUMO to the conduction band.^58^ The HOMO–LUMO gap (*E*_gap_) is thought to be valuable for determining the compound stability and chemical reactivity.^59^ The *E*_gap_ value also determines properties such as electronegativity, ionization potential, electron affinity, softness, hardness, reactivity and stability. Smaller *E*_gap_ would result in higher polarizability, which results in an excellent NLO response.^60^ The results of *E*_HOMO_, *E*_LUMO_ and *E*_gap_ computed at the M06/6-311G(d,p) level are mentioned in Table 1, whereas the *E*_gap_ values of HOMO−1/LUMO+1 and HOMO−2/LUMO+2 are depicted in Table S8.†

**Table 1:** Energies of frontier molecular orbitals (FMOs) of TPR and (TPD1–TPD6)^a^

Systems	*E*_(HOMO)_	*E*_(LUMO)_	*E*_gap_
TPR	−5.744	−2.567	3.177
TPD1	−5.403	−3.28	2.123
TPD2	−5.441	−3.572	1.869
TPD3	−5.437	−3.649	1.788
TPD4	−5.403	−3.361	2.042
TPD5	−5.402	−3.396	2.006
TPD6	−5.398	−3.417	1.981

a Band gap = *E*_LUMO_ − *E*_HOMO_, units in eV.

**Fig. 3:**
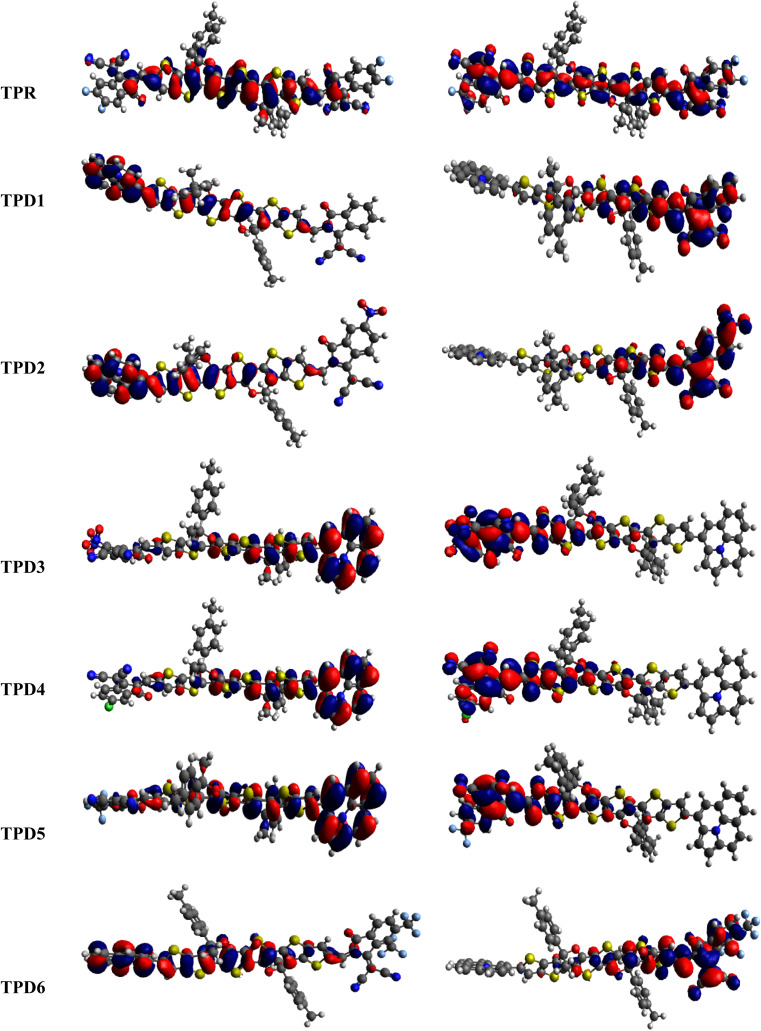
HOMOs and LUMOs of the reference (TPR) and designed compounds (TPD1–TPD6).

The distribution scheme of FMOs plays a significant role in determining the optoelectronic characteristics and conductivity of compounds. In this study, FMO analysis is conducted for TPR and TPD1–TPD6 using the M06 level of theory combined with the 6-311G(d,p) basis.^61–63^Fig. 3 illustrates the distribution arrangement of HOMOs and LUMOs exhibited by FMOs. For TPR, the HOMO and LUMO are determined as −5.744 eV and −2.567 eV, respectively, resulting in *E*_gap_ of 3.177 eV. Similarly, the energy values of the LUMO level are observed as −3.28, −3.572, −3.649, −3.361, −3.396 and −3.417 eV, correspondingly for the respective compounds. These results, along with *E*_gap_, are shown in Table 1. The *E*_gap_ value for TPR and TPD1–TPD6 was calculated as 3.177, 2.123, 1.869, 1.788, 2.042, 2.006 and 1.981 eV, respectively.

In this study, the NFA-based engineered molecules have shown narrower HOMO–LUMO energy gaps as compared to the reference compound (TPR). It has been known from the previous analysis that the electron-withdrawing character and energy gap of compounds are inversely correlated.^64–66^ Molecules with stronger electron-withdrawing end-capped units exhibited a narrower band gap and *vice versa*. TPD1 has a band gap of 2.123 eV owing to the introduction of a donor moiety in the D–π–A molecule, which leads to an increase in the resonance and conjugation. The next derivative (TPD2) shows an *E*_gap_ value of 1.869 eV, which is due to the introduction of a –NO_2_ group present at its terminal acceptor end. Among the designed molecules, TPD3 has shown the lowest energy gap of 1.788 eV, which might be attributed to the strong electron-withdrawing characteristics of two groups (–CN and –NO_2_). TPD4 exhibited an *E*_gap_ value of 2.042 eV because of the introduction of a chlorine (–Cl) group at the terminal position of the acceptor moieties. Similarly, the last two derivatives, *i.e.*, TPD5 and TPD6 exhibited slightly higher values as 2.006 and 1.981 eV. The reason is the presence of –CF_3_ electron-withdrawing groups (one in TPD5 and two in TPD6). Overall, the derivatives exhibited narrower *E*_gap_ compared to TPR, indicating their improved conducting ability. The increasing order of *E*_gap_ for the designed structures, including the reference molecule, is as follows: TPD3 < TPD2 < TPD6 < TPD5 < TPD4 < TPD1 < TPR.

From the above discussion, it is evident that all designed molecules (TPD1–TPD6) might be better NLO candidates for the future, suggesting improved optoelectronic properties compared to TPR. Materials with narrower energy band gaps are more effective for nonlinear optics (NLO).

### Global reactivity parameters (GRPs)

The values of *E*_gap_ of FMOs are crucial to determine the global reactivity parameters such as ionization potential (IP),^67^ global softness (*σ*),^68^ global hardness (*η*),^49^ global electrophilicity index (*ω*),^69^ global electron affinity (EA), electronegativity (*X*)^70^ and chemical potential (*μ*);^71^ it is helpful to know the strength of FMOs (*E*_gap_ = *E*_LUMO_ − *E*_HOMO_). The ionization potential indicates an atom's capacity of donating electrons and is equal to energy required to remove an electron from its HOMO orbital. Additionally, the HOMO/LUMO energy gap had an inverse relationship with reactivity and a direct relationship with the chemical potential, hardness and compound stability. Global reactivity parameters were estimated using following eqn (5)–(12):

5 IP = −*E*_HOMO_

6 EA = −*E*_LUMO_

7
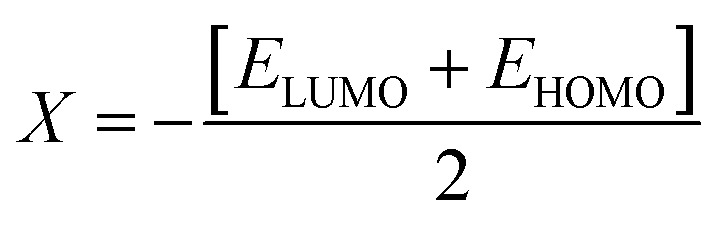

8 *η* = IP − EA

9
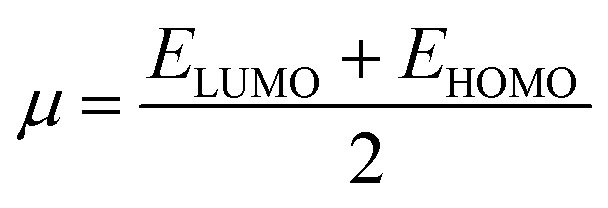
10
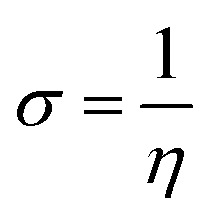
11
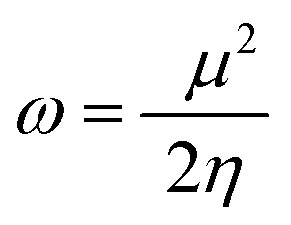
12
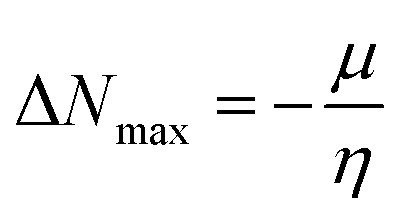


Δ*N*_max_ = −*μ*/*η* indicates the molecule's capacity to absorb additional electrical charge from the surrounding.^72^ The reactivity of atoms and molecules can be indicated by their IP, which refers to how much power is needed to extract one electron from them. Greater stability and chemical inertness are indicated by high ionization energy, whereas lower IP values indicate higher reactivity. However, EA describes the capacity of a molecule to accept the electrons.^73^ The series of compounds show distinct trends in theoretically calculated electron affinity (EA) values. TPD2 and TPD3 exhibit the highest EA, while TPD6 has values lower than those of TPD2 and TPD3 but higher than those of TPD5, TPD4 and TPD1. This observed variation can be attributed to the specific substituents and their positions on the benzene ring. In TPD2 and TPD3, the presence of a nitro group at the *ortho* and *para* positions leads to a substantial increase in EA, reflecting the cumulative effect of two nitro groups.^74^ However, TPD6, with two trifluoromethyl groups at the *ortho* and *para* positions, shows a decrease in EA compared to TPD2 and TPD3, possibly due to the electron-withdrawing nature of trifluoromethyl being less pronounced than that of nitro groups.^75^ However, the presence of chloro and the lack of electron-withdrawing groups in TPD4 and TPD1 derivatives contribute to their lower electron affinity. The electrophilic strength of compounds is often evaluated using the above-mentioned parameters. Additionally, Δ*E* between HOMO and LUMO is directly linked to hardness, chemical potential and compound stability. Conversely, the reactivity is inversely connected to these parameters. Consequently, molecules with smaller energy gaps are considered more reactive and less stable, exhibiting greater polarization. Such molecules are highly competitive in providing superior nonlinear optical responses.^76–78^Table 2 displays that the calculated values for the IP, ranging from 5.403 to 5.398 eV, were lower than that of parent chromophore (5.744 eV). This indicates that these compounds have reduced energy requirement for electron release, making them easily polarizable as compared to TPR. Among the derivatives, TPD2 exhibited the highest IP at 5.441 eV. The decreasing order of IP values is as follows: TPR > TPD2 > TPD3 > TPD4 = TPD1 > TPD5 > TPD6. The *η* and *σ* of designed compounds are linked to their Δ*E* and provide an insight into their reactivity. The *η* is directly related to Δ*E* and inversely related to the reactivity. Similarly, the *σ* values of compounds show an opposite behavior from *η*. A higher Δ*E* value corresponds to greater hardness, resulting in lower ICT and reduced reactivity. The global softness values of the above-mentioned derivatives are obtained in the range of 0.470–0.504 eV^−1^. Notably, TPD3 exhibited the highest *σ* value (0.559 eV^−1^) among all the studied molecules, indicating itself as the most polarizable species. This finding is significant as TPD3 shows promising NLO properties. Materials with higher softness values are more suitable for nonlinear optics (NLO).

**Table 2:** Global reactivity values for TPR and (TPD1–TPD6)^a^

Compounds	IP	EA	*X*	*H*	*M*	*ω*	*Σ*	Δ*N*_max_
TPR	5.744	2.567	4.155	3.177	−4.155	5.435	0.314	2.616
TPD1	5.403	3.28	4.341	2.123	−4.341	8.880	0.470	4.091
TPD2	5.441	3.572	4.506	1.869	−4.506	10.869	0.535	4.824
TPD3	5.437	3.649	4.543	1.788	−4.543	11.542	0.559	5.081
TPD4	5.403	3.361	4.382	2.042	−4.382	9.403	0.489	4.291
TPD5	5.402	3.396	4.399	2.006	−4.399	9.646	0.498	4.399
TPD6	5.398	3.417	4.4075	1.981	−4.4075	9.806	0.504	4.452

a IP = ionization potential, EA = electron affinity, *X* = electronegativity, *μ* = chemical potential, *η* = global hardness, *σ* = global softness, and *ω* = global electrophilicity.

### Density of states (DOS) analysis

**Fig. 4:**
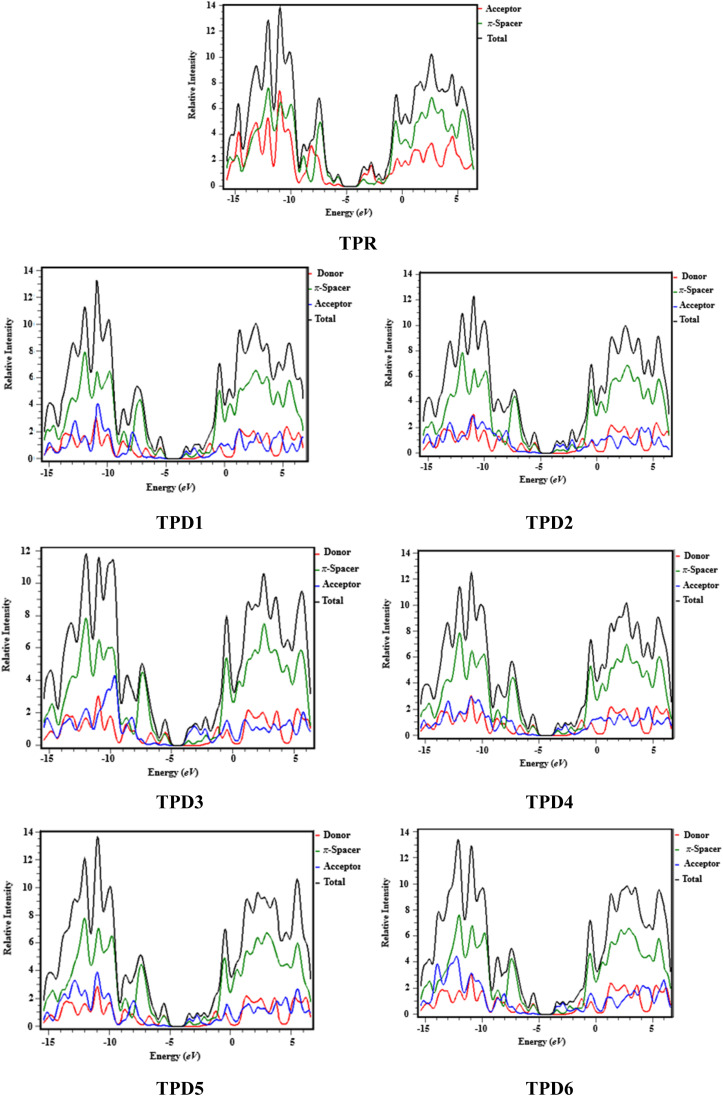
DOS graphs of reference (TPR) and designed compounds (TPD1–TPD6).

The density of states refers to the various electronic states available per unit volume per unit energy that are filled with electrons at specific energy levels (DOSs). By utilizing DOS calculations, one can determine energy difference and overall distribution of energy levels with respect to energy.^79^ The DOS analysis is utilized to determine the electronic properties that supported the FMO study^80^ of TPR and TPD1–TPD6, as shown in Fig. 4. The DOS reveals how the electrons are distributed from HOMO to LUMO. In the DOS pictographs, the energy is represented on the horizontal axis in electron volts (eV), while the vertical axis represents the relative intensity. The negative energy values demonstrate HOMOs, whereas positively charged outcomes represent LUMOs and the distance between them signifies the band gap.^81^ As a result of the fact that all the chromophores contain various acceptor groups with differing strengths and conjugation, the peaks of the plot change between reference and designed molecules. The DOS results confirmed the findings that are shown in the FMO diagrams. By calculating the DOS percentages on the HOMOs and LUMOs of designed compounds, we indicated that how different acceptor moieties affect the distribution of electrons on their molecular orbitals in certain patterns.^82^

In the case of TPR, the acceptor group appears to contribute 19.5% to HOMO and 57.2% to LUMO. Herein, the HOMO is predominantly contributed by the π-spacer, accounting for 80.5%, while the LUMO is primarily influenced by π-spacer with a contribution of 42.8%. For compounds TPD1–TPD6, the HOMO is predominantly influenced by the donor groups with contributions of 48.9, 59.0, 60.9, 51.5, 46.5 and 47.6%, respectively. Similarly, the LUMO is primarily contributed by the donor groups with contributions of 0.4, 0.2, 0.2, 0.3, 0.3 and 0.3%, respectively. The acceptor part has 3.9, 3.4, 2.9, 3.4, 4.0 and 4.1% contributions to HOMO and 59.0, 71.2, 67.5, 58.9, 60.4 and 62.2% contributions to the LUMO in TPD1–TPD6, respectively. In compounds TPD1–TPD6, the π-linker groups exhibit contributions of 47.2, 37.6, 37.1, 45.1, 49.5 and 48.3 to the HOMO. Similarly, these acceptor groups contribute 40.6, 28.6, 32.2, 40.7, 39.3 and 37.5% to LUMO. The observed contributions highlight the significant role of modifying effective acceptor moieties in the design of different compounds, influencing the transmission of electronic charge clouds in various manners in TPD1–TPD6, respectively. The donor group exhibits a maximum charge density of −11 eV in the HOMO of compound TPD1, TPD2, TPD4, TPD5, TPD6 and of −10 eV in TPD3, while the acceptor group displays a maximum charge density of 6 eV in LUMO for compounds TPD1–TPD6 except TPD5, which shows charge density at −4 eV. The extensive analysis of DOS consistently supports and confirms the identical conclusions reached through the scientific investigation of the FMO. The density of states (DOS) is pivotal for nonlinear optics (NLO), as it guides the identification of energy levels crucial for efficient NLO responses, aiding in material selection and predicting electronic transitions essential for enhanced nonlinear optical properties.

### UV-visible investigations

To investigate the impact of bridging core units and acceptor units on spectral characteristics, the absorption spectra^83^ of proposed compounds were obtained through TD-DFT calculations. UV-Vis spectroscopy is utilized to gain insights into the absorption wavelength (*λ*_max_), transition energy (*E*) and oscillator strength (*f*_os_) of substances, with the objective of comprehending their characteristics. The presence of electron-withdrawing groups frequently increases the values of *λ*_max_.^84,85^ Consequently, the observed shift towards longer wavelengths is probably in the absorption values, which is attributed to the incorporation of prolonged conjugated electron-withdrawing groups.^86^

**Fig. 5:**
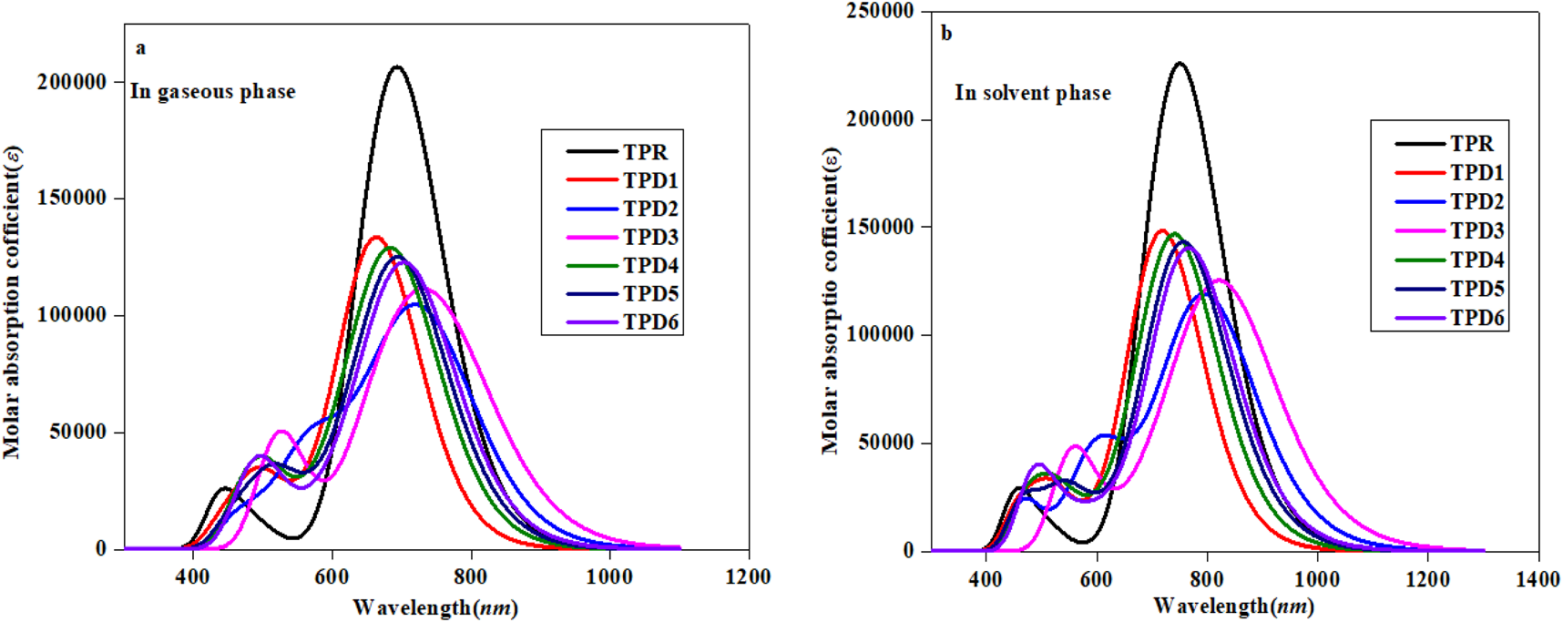
Absorption spectra of the reference (TPR) and designed compounds (TPD1–TPD6) in gaseous (a) and solvent (b) phases.

Table 3 provides a comparison between the theoretical and experimental data for the inspected molecules (TPR and TPD1–TPD6), including their *λ*_max_, *f*_os_, *E* as well as the type of molecular orbital excitations. The corresponding spectra for these molecules are depicted in Fig. 5a and b. It is important to note that each compound exhibited absorption in the ultraviolet (UV) region.

**Table 3:** Wavelength (*λ*), excitation energy (*E*), oscillator strength (*f*_os_) and nature of molecular orbital contributions of compounds (TPR and TPD1–TPD6) in the gaseous phase^a^

Compounds	DFT *λ* (nm)	*E* (eV)	*f*_os_	MO contributions
TPR	692.688	1.790	2.852	H → L (96%), H−1 → L+1 (3%)
TPD1	666.223	1.861	1.785	H → L (86%), H−1 → L (11%)
TPD2	737.167	1.682	1.202	H → L (88%), H−1 → L (11%)
TPD3	765.335	1.620	1.090	H → L (93%), H−1 → L (6%)
TPD4	691.490	1.793	1.631	H → L (88%), H−1 → L (10%)
TPD5	702.579	1.765	1.575	H → L (88%), H−1 → L (10%)
TPD6	711.939	1.742	1.544	H → L (88%), H−1 → L (10%)

a MO = molecular orbital, H = HOMO, L = LUMO, *f*_os_ = oscillator strength, and λ (nm) = wavelength.

Table 3 reveals that the *λ*_max_ values for the entitled compounds (TPR and TPD1–TPD6) lie in the range of 666.223–737.167 nm. Moreover, it is observed that the calculated *λ*_max_ value for all the studied compounds is higher than that of the reference compound (TPR) except TPD1 and TPD4. The *λ*_max_ values for the designed compounds follow a decreasing order as follows: TPD3 (765.335) > TPD2 (737.167) > TPD6 (711.939) > TPD5 (702.579) > TPR (692.688) > TPD4 (691.490) > TPD1 (666.223 nm). Specifically, compound TPD3 demonstrates a *λ*_max_ value of 765.335 nm with the corresponding energy and *f*_os_ values as 1.620 eV and 1.090 for two distinct transitions, indicating their relative strengths and the important transition of HOMO → LUMO (93%) respectively it is worth noting that substituents may have an impact on the absorption wavelength. Among all the designed compounds, TPD1 exhibits an absorption band with a minimum value of 666.223 nm. The corresponding vertical excitation *i.e.*, H → L contributes to approximately 86% of the total transition intensity along with 1.861 eV for energy and 1.785 for *f*_os_.

In the chloroform solvent, compounds under study exhibited higher *λ*_max_ values in comparison to TPR except TPD1 and TPD4 (Table 4). The *λ*_max_ values of all entitled compounds are obtained within the range of 833.619–719.709 nm. The compound (TPD3) displayed the highest *λ*_max_ values of 833.619 nm with a *f*_os_ and *E* value of 1.579 and 1.487 eV, respectively. The declining order of *λ*_max_ of the entitled compounds is as follows: TPD3 (833.619) > TPD2 (801.294) > TPD6 (768.465) > TPD5 (758.731) > TPR (749.602) > TPD4 (743.17) > TPD1 (719.709 nm). Their transition energies (eV) follow thin verse order as: TPD1 (1.723) > TPD4 (1.668) > TPR (1.654) > TPD5 (1.634) > TPD6 (1.613) > TPD2 (1.547) > TPD3 (1.487 eV).

**Table 4:** Wavelength (*λ*), excitation energy (*E*), oscillator strength (*f*_os_), and the nature of molecular orbital contributions of TPR and TPD1–TPD6 in the solvent phase^a^

Compounds	DFT *λ* (nm)	*E* (eV)	*f*_os_	MO contributions
TPR	749.602	1.654	3.120	H → L (95%), H−1 → L+1 (3%)
TPD1	719.709	1.723	2.023	H → L (77%), H−1 → L (19%)
TPD2	801.294	1.547	1.556	H → L (77%), H−1 → L (20%)
TPD3	833.619	1.487	1.579	H → L (82%), H−1 → L (15%)
TPD4	743.177	1.668	1.980	H → L (78%), H−1 → L (18%)
TPD5	758.731	1.634	1.940	H → L (79%), H−1 → L (17%)
TPD6	768.465	1.613	1.901	H → L (80%), H−1 → L (16%)

a MO = molecular orbital, H = HOMO, L = LUMO, *f*_os_ = oscillator strength, and *λ* (nm) = wavelength.

The observed increase in *λ*_max_ and lower excitation energy values indicates enhanced charge transfer capabilities within the chromophores, making them promising candidates for nonlinear optical (NLO) applications. This correlation between UV-Vis parameters and NLO potential is fundamental, as it sheds light on the electronic behavior of material and its ability to respond to external fields, thereby establishing a basis for favorable photo-electronic characteristics in the context of NLO. Among them, TPD3 exhibits minimum transition energy, smallest band gap and the maximum *λ*_max_ value, making it a promising material with favorable photo-electronic characteristics in the field of NLO. In summary, TPD3 can be considered as an acceptable candidate for harnessing its photo-electronic characteristics in NLO.

### Transition density matrix (TDM) and hole electron analysis

The exploration of electronic charge transfer within the investigated compounds, namely, TPR and TPD1–TPD6, has been conducted through a comprehensive analysis utilizing transition density matrix (TDM) and hole–electron analyses. TD-DFT computations employing the M06/6-311G(d,p) functional have provided detailed heat maps for TPR and TPD1–TPD6, revealing valuable insights into electro–hole pair localization, charge excitation phenomena, and interactions between donor (D) and acceptor (A) moieties in excited states.

**Fig. 6:**
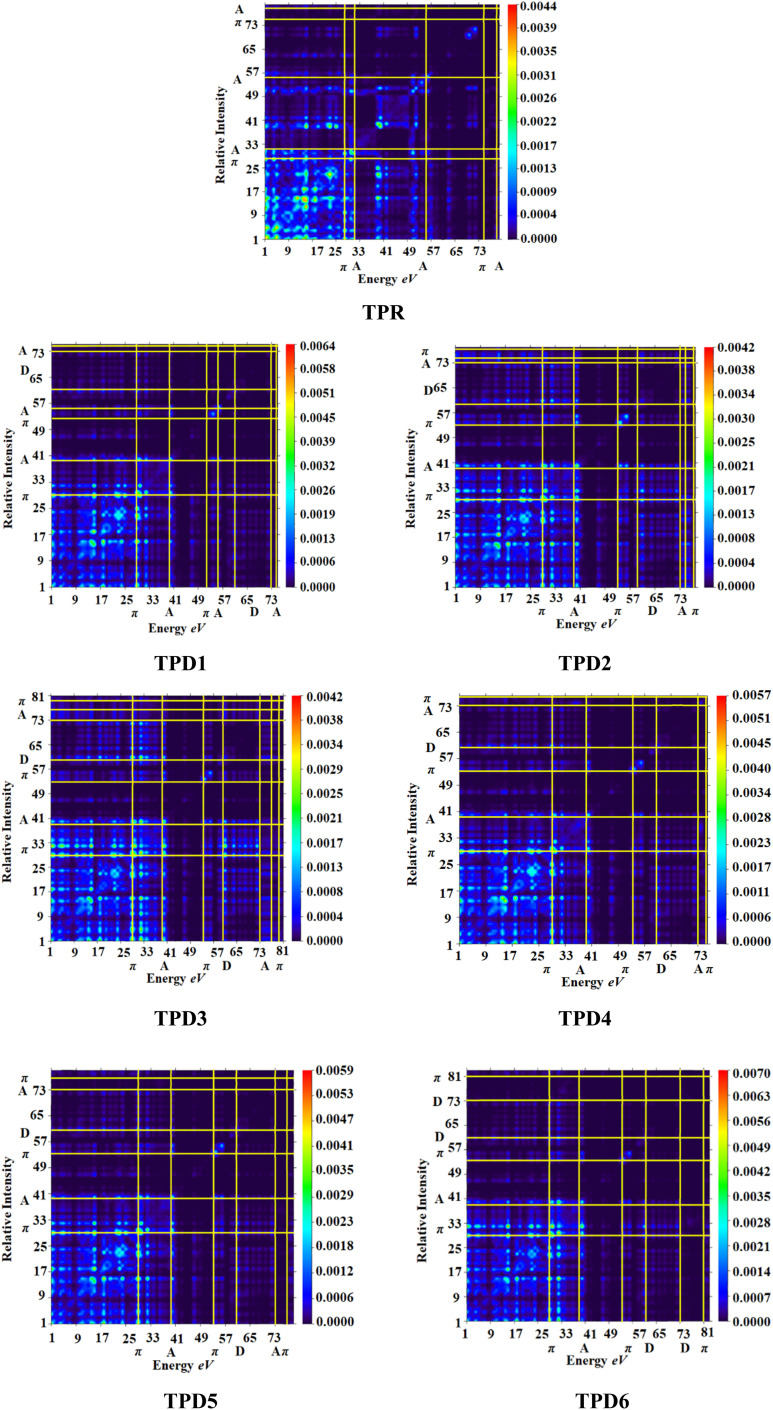
Heat maps of the reference (TPR) and designed compounds (TPD1–TPD6).

The TDM analysis, with a specific focus on the acceptor and π-linker segments, delivers a nuanced understanding of charge density transference. Brightly colored spots in the heat maps denote the flow of charges, illustrating effective diagonal charge transfer coherence within each chromophore. Notably, the higher concentration of the electronic cloud over the π-linker in TPR and TPD1–TPD6, with TPD3 exhibiting denser charges on the acceptor region, suggests enhanced charge transfer efficiency in TPD3. The substantial charge coherence observed in both off-diagonal and diagonal elements across all compounds implies significant exciton dissociation, a pivotal aspect in the engineering of optoelectronic materials. Fig. 6 displays the heat maps representing the electron analysis for TDM analysis.

**Fig. 7:**
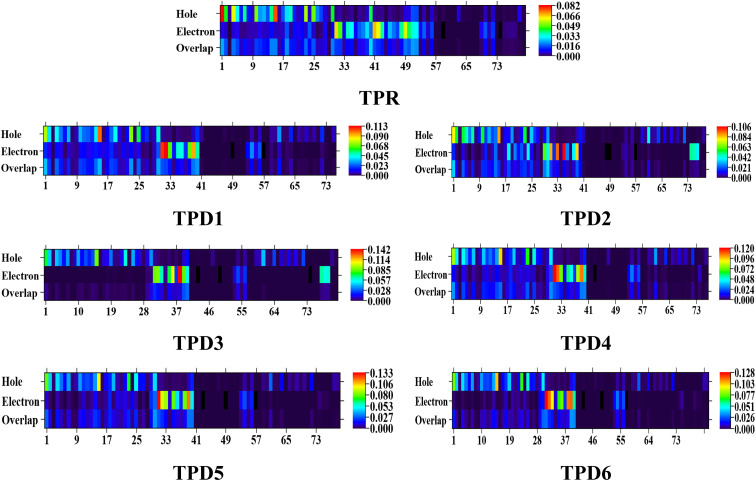
Graphical representation of hole electron analysis of the reference (TPR) and designed compounds (TPD1–TPD6).

Simultaneously, hole–electron analysis, employing Multiwfn 3.7, unveils the charge dynamics within the compounds.^87,88^Fig. 7 depicts heat maps representing electron and hole analyses for TDM, showcasing the efficient charge transfer facilitated by electron-donating groups. This integrative methodology validates findings through density of states (DOS) and frontier molecular orbital (FMO) analyses, providing a thorough understanding of charge transfer phenomena and offering insights into the optimization of material properties, particularly in the realm of nonlinear optical research.^89^

Furthermore, binding energy (*E*_b_), calculated as the difference between energies of electrical and optical bandgaps, serves as a crucial metric for estimating the optoelectronic characteristics of the studied compounds.

According to data mentioned in Table 5, a decreasing order of binding energy values of all the studied compounds is observed as: TPR > TPD1 > TPD4 > TPD5 > TPD6 > TPD2 > TPD3. TPD3 exhibits a higher degree of charge segregation as compared to other designed chromophores, as indicated by its least *E*_b_ value (0.301 eV). A strong correlation between *E*_b_ and the results obtained from TDM analysis provides further support for the suitability of TPD3 as a potential candidate for efficient non-linear optic materials. The consistent behavior observed between the binding energy and TDM results indicates a high level of coherence and reinforces the tremendous potential of TPD3 for delivering optimal performance in terms of charge separation and overall device efficiency.

**Table 5:** Calculated HOMO–LUMO gap (*E*_H_ − *E*_L_), *E*_opt_ first single excitation energies and excitation binding energies (*E*_b_)

Compounds	*E*_H_ − *E*_L_	*E*_opt_	*E*_b_
TPR	3.177	1.654	1.523
TPD1	2.123	1.723	0.400
TPD2	1.869	1.547	0.322
TPD3	1.788	1.487	0.301
TPD4	2.042	1.668	0.374
TPD5	2.006	1.634	0.372
TPD6	1.981	1.613	0.368

### Natural bond orbital (NBO) analysis

The NBO analysis is one of the widely used approaches which is used to calculate the natural charges in the D–π–A based compounds. They also exhibit excellent performance for the interpretation of conjugative interaction and charge transformation phenomena in the system under investigation. Moreover, the information about inter- and intra-molecular H-bonding is provided by the NBO research.^90^ Furthermore, the analysis is beneficial for understanding the consistent representation of charge density transfer from occupied, bonding, or donor Lewis-type NBOs to unoccupied, non-bonding or non-Lewis NBOs. By applying the second-order perturbation theory, we can explore the stabilization energy of molecules and eqn (13) is utilized for this purpose.^91^13
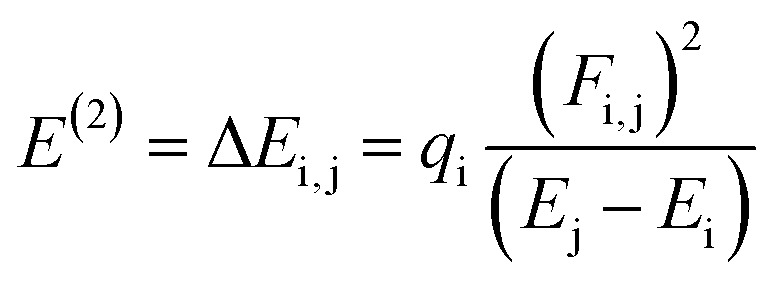


The energy of stabilization, denoted as *E*^(2)^, is influenced by various factors involving donors and acceptors, represented by subscripts i and j. In this context, *E*_i_, *E*_j_, *F*_i,j_ and *q*_i_ correspond to specific components, including diagonal and off-diagonal elements of the NBO Fock matrix, as well as the occupancy of orbitals. The primary outcomes of the NBO analysis are presented in Table 6.

**Table 6:** Representative values of NBO analysis for the studied chromophores (TPR and TPD1–TPD6)

Compounds	Donor (i)	Type	Acceptor (j)	Type	*E*(2) [kcal mol^−1^]	*E*(j) − *E*(i) [a.u.]	*F*(i, j) [a.u.]
TPR	C9–C10	π	C30–C32	π*	28.62	0.31	0.084
	C85–N86	π	C87–N88	π*	0.77	0.47	0.017
	C59–C63	σ	C63–N64	σ*	8.21	1.61	0.103
	C1–C2	σ	C3–O28	σ*	0.5	0.98	0.02
	O28	LP(2)	C1–C2	π*	34.46	0.35	0.106
	O58	LP(2)	C34–C36	σ*	20.82	0.75	0.113
TPD1	C25–C27	π	C31–C33	π*	29.04	0.31	0.085
	C66–N67	π	C68–N69	π*	0.79	0.47	0.017
	C45–C68	σ	C68–N69	σ*	8.18	1.61	0.103
	C20–C21	σ	C21–S23	σ*	0.5	0.94	0.019
	N100	LP (1)	C83–C97	π*	43.95	0.31	0.106
	O44	LP (2)	C34–C41	σ*	20.31	0.76	0.112
TPD2	C25–C27	π	C31–C33	π*	31.48	0.3	0.087
	C66–N67	π	C68–N69	π*	0.78	0.47	0.017
	C45–C68	σ	C68–N69	σ*	8.22	1.61	0.103
	C12–S14	σ	C15–C16	σ*	0.5	1.23	0.022
	N100	LP(1)	C83–C97	π*	43.96	0.31	0.106
	O44	LP(2)	C34–C41	σ*	20.95	0.75	0.113
TPD3	C25–C27	π	C31–C33	π*	31.66	0.3	0.088
	C65–N66	π	C67–N68	π*	0.73	0.48	0.017
	S23–C27	σ	C21–C24	σ*	5.66	1.2	0.074
	C13–S18	σ	C1–C2	σ*	0.51	1.24	0.022
	N99	LP(1)	C82–C96	π*	43.98	0.31	0.106
	O43	LP(2)	C34–C41	σ*	21.65	0.73	0.114
TPD4	C25–C27	π	C31–C33	π*	29.86	0.31	0.086
	C66–N67	π	C68–N69	π*	0.82	0.47	0.018
	C45–C68	σ	C68–N69	σ*	8.15	1.61	0.103
	C12–S14	σ	C15–C16	σ*	0.51	1.23	0.022
	N100	LP(1)	C83–C97	π*	43.97	0.31	0.106
	O44	LP(2)	C34–C41	σ*	20.69	0.75	0.113
TPD5	C25–C27	π	C31–C33	π*	30.59	0.31	0.087
	C66–N67	π	C68–N69	π*	0.76	0.47	0.017
	C45–C68	σ	C68–N69	σ*	8.23	1.61	0.103
	C40–H43	σ	C39–C102	σ*	0.5	0.91	0.019
	N100	LP(1)	C83–C97	π*	44.02	0.31	0.106
	O44	LP(2)	C34–C41	σ*	20.78	0.75	0.113
TPD6	C25–C27	π	C31–C33	π*	30.07	0.31	0.086
	C65–N66	π	C67–N68	π*	0.73	0.48	0.017
	C44–C67	σ	C67–N68	σ*	8.4	1.62	0.105
	C102–F107	σ	C102–F106	σ*	0.51	1.35	0.024
	N99	LP(1)	C82–C96	π*	44.02	0.31	0.106
	O43	LP(2)	C34–C41	σ*	21.31	0.74	0.113

Due to orbital overlapping, there are often four main kinds of transitions in the studied compounds, including *σ* → σ*, π → π*, LP → σ* and LP → π*. In each of these transitions, π → π* are considered to be the most obvious, while *σ* → σ* are less obvious. According to data obtained in Table 5, significant hyper-conjugative interactions among TPR and TPD1–TPD6 are observed to be present for the majority of feasible and consistent π → π* transitions, *i.e.*, π(C9–C10) → π*(C30–C32), π(C25–C27) → π*(C31–C33), π(C25–C27) → π*(C31–C33), π(C25–C2) → π*(C31–C33), π(C25–C2) → π*(C31–C33), π(C25–C2) → π*(C31–C33) and π(C25–C2) → π*(C31–C33) possess the highest transition energies such as 28.6, 29.04, 31.48, 31.66, 29.86, 30.59 and 30.07 kcal mol^−1^ due to stronger interactions between donors and acceptors, respectively. However, the least energy π → π* interactions are π(C85–N86) → π*(C87–N88), (C66–N67) → π*(C68–N69), π(C66–N67) → π*(C68–N69), π(C65–N66) → π*(C67–N68), π(C66–N67) → π*(C68–N69), π(C66–N67) → π*(C68–N69) and π(C65–N66) → π*(C67–N68) obtained with the associated lowest stabilization energy values, *i.e.* 0.77, 0.79, 0.78, 0.73, 0.82, 0.76 and 0.73 kcal mol^−1^, accordingly for entitled compounds.

In feeble σ → σ* transitions, the highest energy of stabilization is obtained as 8.21, 8.18, 8.22, 5.66, 8.15, 8.23 and 8.4 kcal mol^−1^ for *σ*(C59–C63) → σ*(C63–N64), *σ*(C45–C68) → σ*(C68–N69), *σ*(C45–C68) → σ*(C68–N69), *σ*(S23–C27) → σ*(C21–C24), *σ*(C45–C68) → σ*(C68–N69), *σ*(C45–C68) → σ*(C68–N69), and *σ*(C44–C67) → σ*(C67–N68) transitions for TPR and TPD1–TPD6, correspondingly. However, least transition energy values are achieved to be 0.5, 0.5, 0.5, 0.51, 0.51, 0.5 and 0.51 kcal mol^−1^ for subsequently *σ*(C1–C2) → σ*(C3–O28), *σ*(C20–C21) → σ*(C21–S23), *σ*(C12–S14) → σ*(C15–C16), *σ*(C13–S18) → σ*(C1–C2), *σ*(C12–S14) → σ*(C15–C16), *σ*(C40–H43) → σ*(C39–C102), and *σ*(C102–F107) → σ*(C102–F106) transitions in the studied molecules (see Table 5).

The LP → π* transitions, *i.e.*, LP2(O28) → π*(C1–C2), LP1(N100) → π*(C83–C97), LP1(N100) → π*(C83–C97), LP1(N99) → π*(C8–C96), LP1(N100) → π*(C83–C97), LP1(N100) → π*(C83–C97), and LP1(N99) → π*(C8–C96), demonstrate the highest stabilization energies such as 34.46, 43.95, 43.96, 43.98, 43.97, 44.02 and 44.02 kcal mol^−1^ for TPR and TPD1–TPD6, correspondingly. The LP → σ* transitions LP2(O58) → σ*(C34–C36), LP2(O44) → σ*(C34–C41), LP2(O44) → σ*(C34–C41), LP2(O43) → σ*(C34–C41), LP2(O44) → σ*(C34–C41), LP2(O44) → σ*(C34–C41) and LP2(O45) → σ*(C34–C41) exhibited the smallest transition energy values, *i.e.*, 20.82, 20.31, 20.95, 21.65, 20.69, 20.78 and 21.51 kcal mol^−1^, respectively.

The overall study of NBOs of the studied compounds has revealed that both the extended hyper-conjugation and high intramolecular charge mobility rate play a crucial role in stabilizing the studied chromophores, thus improving the charge transport (CT) characteristics that are significant for the NLO response.

### Natural population analysis (NPA)

The dipole moment, chemical reactivity, electrostatic interactions between atoms and molecules, as well as many other features of the chemical system are significantly influenced by the charge dispersion on an atom. The phenomena connecting atomic charge, electrostatic potential on the system outside surfaces and electronegativity equalization modification method that takes place in reactions, as illustrated in the Fig. S3,† can be studied using the Mulliken population analysis (MPA).^92^ Atomic electrical charges have a substantial impact on the structure of molecules and their ability to form bonds.^93^ When highly electronegative elements such as O and N are present, the electron density is unevenly distributed among the benzene rings, according to the Mulliken population statistics.^94^ Our goal is to provide the most thorough account of the distribution of electrons among the compounds in the description and to evaluate the reactivity of the shown charges from a quantum chemical standpoint. The Mulliken population study also demonstrates the homogeneous charge distribution of all hydrogen atoms. The majority of carbon atoms have negative charges due to resonance; however, C-atoms close to electronegative atoms show the positive charges. The sulphur atoms show positive charges in compounds, whereas nitrogen, oxygen and certain carbon atoms have strongly negative charges. NPA analysis is a valuable strategy to acquire idea about the reactivity, as all the derivatives have efficient charge transfer mechanism and thus emerged as effective non-linear optic materials.

### Non-linear optical (NLO) properties

The effectiveness of organic molecules in the field of opto-electronics is greatly enhanced by their structural diversity.^95,96^ The crucial characteristics for achieving NLO properties involved incorporating donor–acceptor functionalities at suitable positions, which are enhanced *via* the extended conjugation.^97^ The second-order hyperpolarizability *γ*_total_ is a key parameter in nonlinear optics (NLO), characterizing the material's nonlinear response to an electric field. It goes beyond the linear relationship described by the first-order hyperpolarizability, representing the material's ability for nonlinear optical effects. *γ*_total_ quantifies higher-order contributions to polarization induced by light, crucial for predicting and optimizing nonlinear processes in applications such as telecommunications and laser technology. Researchers aim to enhance materials with high *γ*_total_ values for improved NLO performance.^98,99^ The computed results for dipole moment (*μ*_total_), average linear polarizability 〈*α*〉 and first- and second-order hyperpolarizabilities, *i.e.*, *β*_total_ and *γ*_total_ of compounds, are displayed in Table 7.

**Table 7:** Representative NLO parameters of the studied compounds (TPR and TPD1–TPD6)^a^

Compounds	*μ*_total_	〈*α*〉 × 10^−22^	*β*_total_ × 10^−27^	*γ*_total_ × 10^−32^
TPR	2.11	2.97	2.93	6.31
TPD1	10.72	2.57	3.56	4.41
TPD2	16.07	2.76	5.34	7.00
TPD3	24.17	2.89	7.24	10.0
TPD4	14.73	2.69	4.44	5.40
TPD5	14.53	2.69	4.68	5.67
TPD6	10.81	2.72	5.04	6.25

a *μ*_tot_ in D, 〈*α*〉, *β*_total_ and *γ*_total_ in esu.

The dipole moment of the molecule is effectively produced by difference in electronegativity. The larger the electronegative difference, the higher will be the dipole moment. TPR exhibited a *μ*_total_ of 2.1114 D, whereas the *μ*_total_ values for TPD1–TPD6 range from 24.173 to 10.724 D. The attainment of the D–π–A configuration and addition of powerful electron-withdrawing units may be responsible for this improvement. In TPD3, the dipole moment is reported to be at its maximum value (24.173 D), which might be due to the existence of strong electron-withdrawing units (–CN and –NO_2_). The dominating values of *μ*_*x*_ (10.070, 15.961, 23.105, 14.040, −14.327 and 10.803 D) for TPD1–TPD6, respectively, are presented in Table S30,† which suggested that the stronger polarity is located along their *x*-axis. In case of TPR, the *z*-axis (*μ*_*z*_ = 1.593 D) exhibits the greater polarizability. Moreover, all the examined chromophores displayed a stronger dipole moment than that of *para*-nitro aniline (*p*-nitroaniline = 4.9662 D).

Another significant parameter which determines the NLO characteristics of organic molecules is 〈*α*〉. The highest value of 〈*α*〉 is 2.89 × 10^−22^ esu obtained for TPD3 with tensor values as 5.84, 0.01 and 1.10 × 10^−22^ esu along the *x*-, *y*- and *z*-axis, respectively. All of the designed chromophores possess comparable 〈*α*〉 values with their reference compound (TPR), which range from 2.57 to 2.97 × 10^−22^ esu.

When it comes to organic chromophores, the effectiveness of CT *via* their respective π-bridges from the donor to the acceptor can be used to predict the NLO behavior. In short, the delocalization of π-electrons coincides with the increase in the hyper-polarizability actions of the mentioned chromophores. The HOMO/LUMO band gap becomes smaller as a result of this delocalization. According to the literature, *E*_gap_ has a significant impact on a molecule's tendency to become polarized. The narrower the band gap, the higher the polarizability values and *vice versa*. In our investigation, the chromophores were also subjected to the same property analysis, and compound TPD3 demonstrated the highest *β*_total_ value of 7.24 × 10^−27^ esu and the narrowest band gap of 1.788 eV. Due to the strong push–pull configuration, all designed derivatives TPD1–TPD6 exhibited excellent *β*_total_ responses (3.56 × 10^−27^ to 7.24 × 10^−27^ esu) in comparison to the TPR (2.93 × 10^−27^ esu). Moreover, a systematic link is seen between the molecular structures and the *β*_total_ values of compounds. The nonlinearity of the chromophore is raised by the strength of linked “A” substituents such as chloro (–Cl), fluoro (–F), nitro (–NO_2_) and cyano (–CN) groups and the total parameter was typically increased with the extension of the conjugated system. The TPR displayed stronger hyperpolarizability values along the *x*- and *z*-axis (*β*_*xxz*_ = 7.00 × 10^−29^ esu); however, other derivatives show higher tensor values along the *x*-axis (*β*_*xxx*_), as shown in Table S32.†

The *γ*_total_ amplitudes for TPR and TPD1–TPD6 are also impacted by the aforementioned characteristics. As indicated in Table S33,† a significant *γ*_total_ response is seen along the *x*-axis. Among all the derivatives, TPD2 has shown the highest value as 7.00 × 10^−32^ esu. The following decreasing order of *γ*_total_ response is obtained for the studied chromophores: TPD2 > TPR > TPD6 > TPD5 > TPD4 > TPD1 > TPD3. The NLO behavior of *p*-NA molecules is utilized as the standard for comparison with our investigated compounds. The reactions of TPR and TPD1–TPD6 to first-order hyper-polarizability were respectively, 0.81 × 10^−4^ esu, 0.986 × 10^−4^ esu, 1.479 × 10^−4^ esu, 2.005 × 10^−4^ esu, 1.229 × 10^−4^ esu, 1.296 × 10^−4^ esu and 1.396 × 10^−4^ esu times larger than those to *p*-NA.

Concluding the above discussion, TPD3 is considered as the most effective derivative for NLO owing to its comparison with *p*-NA (4.9662 D). The above-mentioned reason also leads to the conclusion that various acceptor types with π-conjugations play amazing roles in producing significant amplitudes of NLO.

## Conclusions

A series of non-fullerene organic molecules have been designed by structural tailoring with various acceptor units in order to develop higher efficacy non-linear optic materials. A significant reduction in energy gap (1.788–2.123 eV) between orbitals with a bathochromic shift (666.223–765.335 nm) and higher polarizability has been investigated in the fabricated molecules. The NBO analysis explored that effective electronic clouds are transferred from the donor to the acceptor *via* π-linkers. Promising NLO results were achieved for all derivatives, *i.e.*, the highest amplitude of linear polarizability 〈*α*〉, first (*β*_total_) and second (*γ*_total_) hyperpolarizabilities compared to their reference chromophore. Owing to some unique characteristics of TPD3 such as a lower energy gap (1.788 eV) and a higher *λ*_max_ (765.335 nm), they show significant NLO results [〈*α*〉 = 2.89 × 10^−22^ and *β*_total_ = 7.24 × 10^−27^ esu]. The values were also noted to be the highest, *i.e.*, 2.89 × 10^−22^ and 7.24 × 10^−27^ esu, respectively, as compared to all derivatives. In short, these computational findings suggested that TPD3 has unique NLO properties. This structural modification by utilizing various acceptors has played a protruding role in attaining auspicious NLO responses in compounds. Thus, our study has tempted the experimentalists to synthesize the proposed non-linear optic materials for modern optoelectronic high-tech applications.

## Data availability

All data generated or analyzed during this study are included in this published article and its ESI† files.

## Conflicts of interest

There are no conflicts of interest to declare.

## Supplementary Material

RA-014-D3RA06673H-s001
