# Supplementary material for: Influence of acceptors on the optical nonlinearity of 5H-4-oxa-1,6,9-trithia-cyclopenta[b]-as-indacene-based chromophores with a push–pull assembly: a DFT approach
Source: RSC Adv. 2024 Jan 3;14(2):1169–85. doi: 10.1039/d3ra06673h (PMC10762516; doi:10.1039/d3ra06673h)
Supplement: RA-014-D3RA06673H-s001 [file RA-014-D3RA06673H-s001.pdf]

Electronic Supplementary Material (ESI) for RSC Advances.
This journal is © The Royal Society of Chemistry 2023

Influence of Acceptors on Optical Nonlinearity of 5H-4-Oxa-1,6,9-trithia-cyclopenta[b]-as-indacene Based Chromophores with Push-pull Assembly: A DFT Approach

Muhammad Khalid,[*a,b,] Shahzad Murtaza [a,b] Khansa Gull [a,b] Saba Abid [a,b], Muhammad Imran[c], Ataualpa A.C. Braga,[d]

[a]Institute of Chemistry, Khwaja Fareed University of Engineering & Information Technology, Rahim Yar Khan, 64200, Pakistan

[b]Centre for Theoretical and Computational Research, Khwaja Fareed University of Engineering & Information Technology, Rahim Yar Khan, 64200, Pakistan

[c]Department of Chemistry, Faculty of Science, King Khalid University, P.O. Box 9004, Abha 61413, Saudi Arabia

[d]Departamento de Química Fundamental, Instituto de Química, Universidade de São Paulo, Av. Prof. Lineu Prestes, 748, São Paulo, 05508-000, Brazil

*Corresponding author E-mail addresses:

Dr. Muhammad Khalid (muhammad.khalid@kfueit.edu.pk; khalid@iq.usp.br)

Table S1: Cartesian coordinates of **TPR.**

Atom	X-axis	Y-axis	Z-axis
C	2.058923	-0.53545	-0.60282
C	1.410507	0.673157	-0.41838
C	3.382908	1.980365	-0.80577
C	4.137201	0.683863	-0.61224
C	3.47806	-0.53029	-0.59419
C	5.524382	0.504395	-0.52847
C	5.920945	-0.84056	-0.46143
S	6.882579	1.556399	-0.48569
C	7.29147	-1.04103	-0.38128
C	7.988588	0.165269	-0.38875
H	7.801412	-1.99312	-0.31839
C	0.016846	0.510211	-0.34092
C	-0.37119	-0.81743	-0.45083
S	-1.32027	1.592107	-0.10783
C	-1.76242	-0.97955	-0.36511
C	-2.41414	0.227093	-0.18263
S	4.539384	-1.90897	-0.47549
S	0.964595	-1.90029	-0.68045
C	-3.83255	0.211584	-0.21812
C	-6.26754	0.496493	-0.43838
C	-5.87107	-0.84229	-0.27932

S	-4.89336	1.571282	-0.45541
S	-7.24136	-1.88296	-0.24531
C	-4.48496	-1.00806	-0.14326
C	-7.63654	0.696672	-0.53717
H	-8.13961	1.645742	-0.66311
C	-8.34185	-0.50006	-0.44976
O	2.029653	1.847495	-0.25769
O	-2.39845	-2.14799	-0.51198
C	9.352616	0.502599	-0.29654
C	-3.6854	-2.24718	0.172671
C	10.51046	-0.23835	-0.25308
C	11.84736	0.322942	-0.14527
C	10.64766	-1.69575	-0.43491
C	12.7094	-3.17508	-0.77331
C	12.09627	-1.95905	-0.54841
C	12.80676	-0.76694	-0.40336
C	14.19463	-0.78163	-0.49862
C	14.80916	-1.99459	-0.73432
C	14.08487	-3.1789	-0.86495
H	12.14162	-4.09352	-0.87746
H	14.82136	0.096345	-0.40746
H	9.503257	1.578864	-0.27806
C	-9.70908	-0.83241	-0.52145
H	-9.87015	-1.90596	-0.46682
C	-10.859	-0.08434	-0.61615
C	-10.9824	1.383968	-0.53393
C	-12.203	-0.63736	-0.68917
C	-13.1508	0.477908	-0.5091
C	-14.5387	0.514567	-0.41843
C	-15.1402	1.747419	-0.26781
C	-14.4032	2.929847	-0.2161
C	-13.0278	2.904616	-0.30357
C	-12.428	1.669216	-0.44398
H	-15.1755	-0.35997	-0.44962
H	-12.4495	3.82134	-0.26104
O	-10.109	2.226665	-0.51799
O	9.781874	-2.54368	-0.50363
C	12.20566	1.604571	0.20303
C	-12.5749	-1.93538	-0.94987
C	11.28546	2.618125	0.587153
N	10.57124	3.465423	0.916487
C	13.55688	2.039905	0.263672
N	14.6429	2.430894	0.320201

C	4.036349	3.082747	-0.00092
C	4.65477	4.178332	-0.59004
C	4.088319	2.954625	1.387626
C	5.32779	5.111276	0.189403
H	4.633854	4.311729	-1.66682
C	4.753765	3.88821	2.158825
H	3.606006	2.104607	1.863404
C	5.399735	4.977943	1.571048
H	5.8128	5.957753	-0.29029
H	4.786107	3.76995	3.239128
C	-3.42198	-2.38889	1.667203
C	-2.37149	-3.19529	2.104847
C	-4.23683	-1.78609	2.617269
C	-2.1436	-3.38334	3.456562
H	-1.72298	-3.67539	1.376854
C	-4.00817	-1.98561	3.972854
H	-5.06089	-1.14865	2.30732
C	-2.95827	-2.78131	4.416267
H	-1.31616	-4.01095	3.779812
H	-4.65819	-1.50651	4.700814
C	-11.6643	-2.98262	-1.25796
N	-10.9572	-3.85775	-1.52303
C	-13.9306	-2.35962	-0.98663
N	-15.0206	-2.74187	-1.02132
C	-2.68865	-2.97665	5.87564
H	-3.53773	-2.65888	6.486271
H	-2.47574	-4.02578	6.103698
H	-1.81519	-2.39643	6.194681
C	6.174512	5.950267	2.403571
H	6.248194	6.926272	1.916429
H	5.718151	6.08846	3.387933
H	7.196616	5.588656	2.567411
F	16.12922	-2.05326	-0.84176
F	14.7488	-4.30452	-1.08274
F	-16.4597	1.827201	-0.16808
F	-15.0549	4.07481	-0.07666
C	3.240885	2.275247	-2.28742
H	2.703483	1.456597	-2.77478
H	2.685941	3.204618	-2.44447
H	4.223795	2.356099	-2.76146
C	-4.30878	-3.50886	-0.38705
H	-5.23312	-3.74543	0.147711
H	-4.52277	-3.39633	-1.45393

H	-3.62133	-4.34698	-0.24692

Table S2: Cartesian coordinates of **TPD1.**

Atom	X-axis	Y-axis	Z-axis
C	2.548384	0.705207	-0.04115
C	2.005199	-0.47588	-0.49857
C	4.019841	-1.67593	-0.19488
C	4.720782	-0.3371	-0.18365
C	3.969964	0.806071	-0.01607
C	6.097042	-0.02626	-0.2599
C	6.374456	1.328517	-0.13648
S	7.568667	-0.93697	-0.41283
C	7.746843	1.648489	-0.16265
C	8.529341	0.536299	-0.31152
H	8.156428	2.646812	-0.06544
C	0.604205	-0.42086	-0.57159
C	0.099928	0.813892	-0.18909
S	-0.62855	-1.53232	-1.0754
C	-1.29948	0.879952	-0.28921
C	-1.84096	-0.30923	-0.74724
S	4.921733	2.264705	0.05334
S	1.341966	1.920774	0.310766
C	-3.25216	-0.42132	-0.77772
C	-5.65814	-0.95761	-0.76186
C	-5.38286	0.405961	-0.5689
S	-4.18738	-1.88307	-0.94412
S	-6.83522	1.305885	-0.37004
C	-4.01662	0.715546	-0.58189
C	-7.00262	-1.29145	-0.74578
H	-7.42264	-2.28033	-0.86532
C	-7.8114	-0.17364	-0.54619
O	2.749912	-1.52431	-0.89133
O	-2.02483	1.941347	0.080655
C	-3.35944	2.076713	-0.51226
C	-9.19993	0.010958	-0.41396
H	-9.45416	1.050497	-0.2225
C	-10.2816	-0.83723	-0.49515
C	-10.2799	-2.25605	-0.90215
C	-11.6649	-0.43588	-0.30009
C	-12.5194	-1.5639	-0.72213
C	-13.8994	-1.71106	-0.82028
C	-14.4069	-2.9362	-1.24087
C	-13.5723	-4.00455	-1.55478

C	-12.1935	-3.86261	-1.46141
C	-11.6956	-2.64324	-1.05145
H	-14.5854	-0.90713	-0.58928
H	-11.5138	-4.67405	-1.70221
O	-9.33383	-2.99128	-1.10523
C	-12.1385	0.736548	0.24617
C	3.744474	-2.18468	1.215156
C	2.684093	-3.05814	1.445199
C	4.576831	-1.8553	2.27899
C	2.460859	-3.5783	2.709565
H	2.021686	-3.32655	0.626476
C	4.354077	-2.38713	3.541059
H	5.410315	-1.17382	2.126268
C	3.290248	-3.25207	3.781026
H	1.623307	-4.25305	2.871173
H	5.018748	-2.12	4.35924
C	-4.12467	2.985048	0.425017
C	-4.79212	4.120621	-0.01539
C	-4.22127	2.634699	1.772515
C	-5.55908	4.874293	0.864774
H	-4.7336	4.424707	-1.05533
C	-4.98011	3.391417	2.645269
H	-3.69757	1.75312	2.132822
C	-5.67502	4.519039	2.203393
H	-6.0803	5.75593	0.500363
H	-5.04602	3.102771	3.691494
C	-11.3126	1.759003	0.78853
N	-10.6778	2.607294	1.251786
C	-13.5224	1.030802	0.376956
N	-14.6357	1.316307	0.500437
C	-6.54105	5.30156	3.139925
H	-6.10256	5.355208	4.14073
H	-6.70283	6.320521	2.778405
H	-7.52542	4.829581	3.242304
C	3.026674	-3.79353	5.15179
H	2.322169	-3.15465	5.697247
H	2.587683	-4.79458	5.108953
H	3.943804	-3.84584	5.745181
C	9.977822	0.503375	-0.48107
C	10.76565	-0.54905	0.085191
C	10.60353	1.488638	-1.19624
C	12.02061	1.512931	-1.399
H	10.00714	2.269314	-1.65997

C	12.78317	0.461559	-0.85565
C	12.7059	2.496528	-2.12058
H	12.14479	3.318243	-2.55602
C	14.08258	2.422066	-2.27736
H	14.59633	3.195799	-2.83932
C	10.53328	-1.67301	0.87611
H	9.570898	-1.98304	1.255772
C	11.75757	-2.3128	1.10212
H	11.91986	-3.21321	1.676939
C	12.75416	-1.58538	0.460697
C	14.16205	-1.68513	0.313876
H	14.67176	-2.52178	0.779514
C	14.84829	-0.74939	-0.38997
H	15.92596	-0.82526	-0.49966
C	14.18078	0.367715	-1.00148
C	14.81642	1.37877	-1.72955
H	15.894	1.335425	-1.85911
N	12.13057	-0.51087	-0.15075
H	-14.0035	-4.94688	-1.87602
H	-15.4819	-3.05745	-1.32486
C	4.761872	-2.72272	-1.00108
H	4.987939	-2.35018	-2.00461
H	5.692034	-3.00475	-0.49941
H	4.142722	-3.62024	-1.08224
C	-3.17704	2.630385	-1.91376
H	-4.14391	2.721178	-2.41813
H	-2.69252	3.610675	-1.88283
H	-2.55638	1.947967	-2.50133

Table S3: Cartesian coordinates of **TPD2.**

Atom	X-axis	Y-axis	Z-axis
C	3.125793	0.793747	-0.25946
C	2.513309	-0.42965	-0.43275
C	4.47695	-1.63599	0.095247
C	5.238881	-0.3694	-0.21123
C	4.550886	0.821835	-0.28813
C	6.627466	-0.15865	-0.35906
C	6.977312	1.174408	-0.53402
S	8.047209	-1.15795	-0.30292
C	8.364721	1.406314	-0.62504
C	9.084921	0.247987	-0.52154
H	8.827578	2.378574	-0.74387
C	1.115659	-0.31654	-0.48757

C	0.680513	0.995605	-0.36389
S	-0.17924	-1.4468	-0.71918
C	-0.71724	1.109455	-0.43547
C	-1.32467	-0.12239	-0.62817
S	5.578598	2.206994	-0.54271
S	1.989905	2.117774	-0.14624
C	-2.73547	-0.17457	-0.59263
C	-5.15626	-0.59793	-0.43276
C	-4.82374	0.76946	-0.47603
S	-3.7281	-1.60262	-0.48522
S	-6.23511	1.750726	-0.39838
C	-3.45132	1.012414	-0.57812
C	-6.50653	-0.86897	-0.34771
H	-6.96113	-1.84811	-0.30599
C	-7.27332	0.300007	-0.32394
O	3.193114	-1.57813	-0.59433
O	-1.38005	2.255924	-0.26065
C	-2.7517	2.337239	-0.77826
C	-8.64633	0.544084	-0.2041
H	-8.87055	1.60622	-0.14168
C	-9.75682	-0.28171	-0.17842
C	-9.79785	-1.72339	-0.47082
C	-11.1187	0.174604	0.008031
C	-12.0189	-0.96119	-0.29837
C	-13.4048	-1.07403	-0.34755
C	-13.9603	-2.30707	-0.66276
C	-13.1408	-3.39604	-0.91778
C	-11.7583	-3.31315	-0.87525
C	-11.2296	-2.08173	-0.56515
H	-14.0644	-0.23906	-0.15663
H	-11.1273	-4.17037	-1.07836
O	-8.88475	-2.50643	-0.63629
C	-11.5538	1.401118	0.459635
C	4.218878	-1.79528	1.589031
C	3.186902	-2.62594	2.024214
C	5.023952	-1.18252	2.541407
C	2.965657	-2.82734	3.375469
H	2.548189	-3.11543	1.293855
C	4.802622	-1.39533	3.896183
H	5.833253	-0.52612	2.231421
C	3.769652	-2.2147	4.337082
H	2.151776	-3.47373	3.696447
H	5.444414	-0.90782	4.626052

C	-3.42856	3.412175	0.04347
C	-4.0937	4.48835	-0.52898
C	-3.44583	3.279288	1.433213
C	-4.77634	5.399919	0.268306
H	-4.1013	4.624307	-1.60559
C	-4.11981	4.191417	2.221301
H	-2.92701	2.443196	1.895006
C	-4.80912	5.264363	1.650332
H	-5.29686	6.232535	-0.19799
H	-4.12408	4.071485	3.302012
C	-10.6899	2.443322	0.893678
N	-10.0252	3.311745	1.268779
C	-12.9265	1.744109	0.587755
N	-14.0301	2.067364	0.703848
C	-5.57653	6.219255	2.509347
H	-5.00366	6.506092	3.396625
H	-5.84145	7.127468	1.962025
H	-6.50794	5.761593	2.862156
C	3.506344	-2.42243	5.796127
H	2.63181	-1.84825	6.123252
H	3.298952	-3.47412	6.017417
H	4.35632	-2.10529	6.406014
C	10.52978	0.103516	-0.66169
C	11.26121	-0.82487	0.146092
C	11.20708	0.864101	-1.57579
C	12.62453	0.772895	-1.756
H	10.65309	1.545164	-2.21578
C	13.33173	-0.15598	-0.96888
C	13.36198	1.527256	-2.6751
H	12.84493	2.249683	-3.29996
C	14.73434	1.354883	-2.78567
H	15.28859	1.950964	-3.50396
C	10.97026	-1.71964	1.174743
H	9.992219	-1.88889	1.600164
C	12.16077	-2.34217	1.566947
H	12.2756	-3.0884	2.339839
C	13.19541	-1.8318	0.790997
C	14.59805	-2.02585	0.698015
H	15.06383	-2.7507	1.356852
C	15.33306	-1.31448	-0.19403
H	16.4067	-1.46153	-0.26362
C	14.72416	-0.34452	-1.06297
C	15.41313	0.436889	-1.99619

H	16.48821	0.316095	-2.09317
N	12.62808	-0.90546	-0.06799
H	-15.0346	-2.43297	-0.71517
N	-13.7651	-4.69039	-1.25203
O	-13.0254	-5.61861	-1.5004
O	-14.9763	-4.74824	-1.25867
C	5.145489	-2.87386	-0.46689
H	5.360092	-2.74834	-1.53225
H	6.075694	-3.08064	0.07004
H	4.4863	-3.73571	-0.33418
C	-2.65071	2.643113	-2.26143
H	-3.64579	2.687929	-2.71457
H	-2.13472	3.593371	-2.42602
H	-2.09279	1.847656	-2.76376

Table S4: Cartesian coordinates of **TPD3.**

Atom	X-axis	Y-axis	Z-axis
C	3.328904	-0.86055	-0.79908
C	2.752538	0.314336	-0.36175
C	4.786904	1.561824	-0.54645
C	5.479008	0.217456	-0.61734
C	4.751567	-0.93217	-0.82307
C	6.862332	-0.0586	-0.58941
C	7.172722	-1.39808	-0.77839
S	8.303185	0.887372	-0.38619
C	8.553666	-1.67999	-0.76996
C	9.302885	-0.55213	-0.56934
H	8.992014	-2.65997	-0.91588
C	1.352228	0.220052	-0.30399
C	0.884821	-1.02968	-0.6893
S	0.085112	1.296314	0.193946
C	-0.50943	-1.13225	-0.60339
C	-1.09125	0.039531	-0.14147
S	5.739058	-2.36257	-0.98262
S	2.160862	-2.1079	-1.1642
C	-2.50094	0.114478	-0.16113
C	-4.91252	0.591104	-0.29217
C	-4.59906	-0.77606	-0.43601
S	-3.47262	1.556548	-0.08175
S	-6.03155	-1.71177	-0.63064
C	-3.23201	-1.05307	-0.35196
C	-6.25805	0.893701	-0.34719
H	-6.69936	1.87746	-0.26512

C	-7.04304	-0.24931	-0.52773
O	3.451818	1.392313	0.030101
O	-1.21412	-2.19893	-0.99286
C	-2.50693	-2.37509	-0.33527
C	-8.41878	-0.4486	-0.68191
H	-8.67863	-1.49066	-0.8579
C	-9.50586	0.402191	-0.61008
C	-9.53811	1.802252	-0.18373
C	-10.8735	-0.00044	-0.88121
C	-11.7557	1.001219	-0.23386
C	-13.0883	1.036816	0.15355
C	-13.5993	2.218922	0.691971
C	-12.8291	3.353997	0.856097
C	-11.4765	3.300652	0.552512
C	-10.9705	2.125947	0.045177
H	-10.8179	4.142505	0.735735
O	-8.62881	2.582456	0.00962
C	-11.2687	-0.97004	-1.76858
C	5.512102	2.486497	0.407816
C	6.16512	3.635816	-0.01584
C	5.577114	2.142271	1.759283
C	6.877941	4.417398	0.886854
H	6.139574	3.9304	-1.05988
C	6.27959	2.926457	2.653771
H	5.072794	1.243711	2.104649
C	6.947567	4.078605	2.232111
H	7.390604	5.308695	0.533911
H	6.322223	2.641961	3.702567
C	-2.25433	-2.87303	1.082735
C	-1.23239	-3.79273	1.315363
C	-3.06177	-2.49361	2.147552
C	-1.02373	-4.30812	2.582478
H	-0.59171	-4.10298	0.494196
C	-2.85364	-3.02176	3.414857
H	-3.86476	-1.77673	1.99607
C	-1.83074	-3.93245	3.656374
H	-0.21836	-5.02053	2.746015
H	-3.49864	-2.71489	4.234551
C	-10.3379	-1.77337	-2.4861
N	-9.60869	-2.43614	-3.08929
C	-12.6047	-1.14646	-2.21627
N	-13.6619	-1.29069	-2.65861
C	-1.58277	-4.47888	5.027811

H	-2.45604	-4.3529	5.673144
H	-1.33155	-5.54334	4.99347
H	-0.74133	-3.9661	5.508204
C	7.699519	4.923102	3.212486
H	8.341346	5.648954	2.706749
H	7.011506	5.479968	3.858815
H	8.325853	4.308663	3.866892
C	10.75199	-0.48534	-0.41204
C	11.50198	0.616273	-0.93488
C	11.41478	-1.48371	0.249846
C	12.83382	-1.47273	0.441065
H	10.84719	-2.30402	0.680204
C	13.55799	-0.37192	-0.05554
C	13.55632	-2.46738	1.109022
H	13.02565	-3.32738	1.507171
C	14.93116	-2.35536	1.260835
H	15.474	-3.13829	1.781139
C	11.22854	1.766199	-1.67345
H	10.25438	2.060528	-2.03457
C	12.4301	2.45444	-1.87758
H	12.5595	3.384719	-2.41176
C	13.45374	1.731686	-1.27469
C	14.85847	1.870842	-1.13092
H	15.33733	2.744588	-1.55989
C	15.57972	0.926634	-0.47498
H	16.65511	1.032286	-0.3676
C	14.95295	-0.23933	0.086442
C	15.6264	-1.26277	0.760832
H	16.70291	-1.19037	0.886085
N	12.86908	0.61106	-0.70911
H	-13.2871	4.24233	1.275165
N	-13.9602	-0.14929	0.069096
O	-13.4911	-1.18357	0.487006
O	-15.0563	0.000223	-0.4105
N	-14.9633	2.274794	1.256981
O	-15.6134	3.263396	1.012807
O	-15.301	1.351551	1.961456
C	4.613873	2.11979	-1.94781
H	4.011356	1.429323	-2.54498
H	4.112794	3.091919	-1.91883
H	5.584597	2.228708	-2.44115
C	-3.20316	-3.43737	-1.16113
H	-4.13921	-3.73976	-0.68247

H	-3.41129	-3.07037	-2.17046
H	-2.56482	-4.32232	-1.22679

Table S5: Cartesian coordinates of **TPD4.**

Atom	X-axis	Y-axis	Z-axis
C	2.597233	-0.89426	-0.83077
C	2.00808	0.246619	-0.32894
C	4.025134	1.531743	-0.4365
C	4.732275	0.204002	-0.59667
C	4.021949	-0.94305	-0.8647
C	6.120315	-0.04787	-0.59747
C	6.453828	-1.36719	-0.86921
S	7.543939	0.914465	-0.35568
C	7.8409	-1.62025	-0.89426
C	8.569841	-0.49042	-0.63829
H	8.297056	-2.5805	-1.10352
C	0.608729	0.136209	-0.27796
C	0.150851	-1.09329	-0.73138
S	-0.66424	1.177273	0.274206
C	-1.24533	-1.20603	-0.65547
C	-1.83152	-0.06454	-0.13436
S	5.03386	-2.34337	-1.11743
S	1.438674	-2.13181	-1.26283
C	-3.24393	0.004764	-0.14244
C	-5.65704	0.477433	-0.21478
C	-5.34364	-0.8761	-0.43629
S	-4.21886	1.436327	0.027038
S	-6.78068	-1.80203	-0.64632
C	-3.97082	-1.15149	-0.38812
C	-7.00658	0.779831	-0.22192
H	-7.44587	1.756277	-0.0769
C	-7.79415	-0.34916	-0.44479
O	2.691428	1.310614	0.124404
O	-1.94922	-2.25362	-1.10058
C	-3.23897	-2.46883	-0.44717
C	-9.17865	-0.56388	-0.55568
H	-9.41048	-1.60692	-0.75228
C	-10.2796	0.26111	-0.45403
C	-10.288	1.695849	-0.11705
C	-11.6618	-0.16533	-0.61185
C	-12.5239	0.992875	-0.30036
C	-13.9058	1.15421	-0.2492
C	-14.4239	2.401253	0.074261

C	-13.5794	3.473332	0.340361
C	-12.2003	3.336006	0.297544
C	-11.7082	2.089842	-0.02167
H	-14.5945	0.344598	-0.44865
H	-11.5312	4.164333	0.505557
O	-9.35333	2.449637	0.066333
C	-12.1412	-1.3932	-1.01218
C	4.738607	2.392983	0.5831
C	5.344438	3.598824	0.259162
C	4.837879	1.928036	1.896069
C	6.041938	4.320122	1.222606
H	5.292347	3.988737	-0.75241
C	5.527066	2.649709	2.850024
H	4.371312	0.983112	2.162345
C	6.145412	3.86121	2.529079
H	6.514221	5.260161	0.948571
H	5.597313	2.270786	3.867119
C	-2.97833	-3.04056	0.941354
C	-1.96639	-3.98499	1.114644
C	-3.76357	-2.70047	2.035263
C	-1.74936	-4.56616	2.351063
H	-1.33904	-4.26087	0.271096
C	-3.54624	-3.29393	3.272492
H	-4.55528	-1.9627	1.931052
C	-2.53703	-4.23255	3.453385
H	-0.9522	-5.2968	2.468995
H	-4.17322	-3.01623	4.116177
C	-11.329	-2.49982	-1.37925
N	-10.7085	-3.42405	-1.69161
C	-13.5267	-1.6866	-1.12922
N	-14.6409	-1.9742	-1.23845
C	-2.28293	-4.86002	4.788595
H	-3.09001	-4.64402	5.493294
H	-2.18447	-5.94698	4.703658
H	-1.34974	-4.48823	5.226834
C	6.884098	4.634627	3.575796
H	7.415413	5.487138	3.145162
H	6.196825	5.018186	4.3383
H	7.613378	4.002948	4.093321
C	10.01929	-0.39599	-0.49874
C	10.73102	0.74738	-0.98389
C	10.71846	-1.4027	0.110616
C	12.13929	-1.3579	0.286291

H	10.17974	-2.25649	0.512191
C	12.8251	-0.21601	-0.17054
C	12.89881	-2.35658	0.905806
H	12.39864	-3.24812	1.272938
C	14.27101	-2.20838	1.050557
H	14.84237	-2.99477	1.533807
C	10.41493	1.918989	-1.66978
H	9.427209	2.199349	-2.00424
C	11.59353	2.649518	-1.85972
H	11.68941	3.605018	-2.35505
C	12.6453	1.93216	-1.30021
C	14.0467	2.107274	-1.16501
H	14.49454	3.011941	-1.56216
C	14.8026	1.159707	-0.55436
H	15.87541	1.293312	-0.45235
C	14.21686	-0.04661	-0.03574
C	14.92775	-1.07529	0.59044
H	16.00275	-0.97509	0.709453
N	12.10044	0.772456	-0.77542
H	-15.4974	2.545442	0.121423
Cl	-14.2731	5.019768	0.736546
C	-3.93475	-3.4878	-1.32635
H	-4.86937	-3.81754	-0.86351
H	-4.14597	-3.06763	-2.31404
H	-3.29444	-4.36626	-1.44034
C	3.846888	2.182677	-1.79608
H	3.254328	1.526315	-2.44019
H	3.330992	3.14279	-1.70131
H	4.816287	2.339823	-2.27912

Table S6: Cartesian coordinates of **TPD5.**

Atom	X-axis	Y-axis	Z-axis
C	-3.17464	1.019974	0.059732
C	-2.57935	-0.22296	0.010696
C	-4.58769	-1.33591	-0.67015
C	-5.30364	-0.07455	-0.23796
C	-4.59888	1.074012	0.037521
C	-6.69366	0.151765	-0.14771
C	-7.03189	1.455912	0.184681
S	-8.11514	-0.81655	-0.38047
C	-8.41989	1.696969	0.246377
C	-9.14527	0.571822	-0.03725
H	-8.87872	2.653266	0.467347

C	-1.18068	-0.13858	0.11564
C	-0.73315	1.167004	0.256955
S	0.103826	-1.30448	0.147549
C	0.662595	1.25048	0.378902
C	1.260788	0.000708	0.334918
S	-5.61619	2.441534	0.414813
S	-2.02571	2.326644	0.229374
C	2.673311	-0.05005	0.296843
C	5.095118	-0.43367	0.039772
C	4.764479	0.892096	0.375726
S	3.663724	-1.42395	-0.11555
S	6.175158	1.868913	0.496018
C	3.39195	1.111193	0.532618
C	6.446867	-0.67664	-0.11849
H	6.904901	-1.62112	-0.37651
C	7.209891	0.472926	0.098991
O	-3.25937	-1.37992	-0.05715
O	1.333416	2.404148	0.445505
C	2.690628	2.366629	1.000428
C	8.581576	0.747862	0.014109
H	8.800186	1.802217	0.167453
C	9.695121	-0.04339	-0.18837
C	9.75869	-1.51473	-0.17556
C	11.05091	0.460181	-0.28175
C	11.9658	-0.69509	-0.15839
C	13.34858	-0.79019	-0.08119
C	13.92425	-2.05232	0.030501
C	13.14128	-3.19872	0.05526
C	11.75382	-3.11346	-0.02293
C	11.19604	-1.86056	-0.12157
H	13.99144	0.079275	-0.09239
H	11.12355	-3.99804	-0.00091
O	8.853761	-2.32473	-0.17602
C	11.45916	1.752386	-0.52597
C	-5.30767	-2.55655	-0.13794
C	-5.92727	-3.48429	-0.96348
C	-5.40785	-2.72213	1.245291
C	-6.64262	-4.54644	-0.41976
H	-5.87344	-3.38669	-2.04293
C	-6.11649	-3.77947	1.78001
H	-4.92662	-2.00275	1.902673
C	-6.75311	-4.71016	0.954532
H	-7.12636	-5.25953	-1.0825

H	-6.18859	-3.89072	2.859505
C	3.384475	3.591921	0.446606
C	4.038338	4.51288	1.254638
C	3.4326	3.765826	-0.93763
C	4.745161	5.568724	0.690675
H	4.018845	4.413151	2.334948
C	4.13318	4.819096	-1.49292
H	2.918875	3.056539	-1.58138
C	4.815998	5.733083	-0.68715
H	5.256848	6.275666	1.338833
H	4.164302	4.936018	-2.57349
C	10.5705	2.827391	-0.80215
N	9.881624	3.72385	-1.04412
C	12.82492	2.138577	-0.58532
N	13.92329	2.494518	-0.63817
C	5.621385	6.836236	-1.29824
H	5.112675	7.270961	-2.1639
H	5.820233	7.633904	-0.57783
H	6.589574	6.459428	-1.64858
C	-7.5209	-5.84875	1.549408
H	-8.02363	-6.43972	0.779782
H	-6.85983	-6.51852	2.110512
H	-8.27986	-5.48856	2.251963
C	-10.5977	0.435565	-0.00192
C	-11.2809	-0.36047	-0.97596
C	-11.3272	1.0699	0.966789
C	-12.7539	0.969105	1.046854
H	-10.8111	1.648388	1.728138
C	-13.4133	0.166709	0.095291
C	-13.5445	1.592907	2.018191
H	-13.0648	2.214914	2.768378
C	-14.9212	1.418927	2.022085
H	-15.5173	1.911967	2.783617
C	-10.9289	-1.1032	-2.10182
H	-9.92527	-1.21942	-2.48309
C	-12.0945	-1.651	-2.6496
H	-12.1636	-2.27876	-3.52628
C	-13.1738	-1.24498	-1.87232
C	-14.5799	-1.4351	-1.89252
H	-15.0059	-2.05399	-2.67494
C	-15.3667	-0.84982	-0.95407
H	-16.4429	-0.99344	-0.97115
C	-14.8091	-0.01935	0.078705

C	-15.5517	0.62829	1.070864
H	-16.6308	0.504901	1.085243
N	-12.6579	-0.45557	-0.85826
H	15.00231	-2.1399	0.100607
C	13.75729	-4.55794	0.178895
F	13.43835	-5.33077	-0.8658
F	13.3151	-5.19077	1.272164
F	15.08653	-4.51686	0.253069
C	2.557979	2.346136	2.512273
H	2.035446	3.239206	2.867042
H	1.993832	1.461299	2.82078
H	3.544627	2.294509	2.982772
C	-4.39933	-1.32474	-2.17628
H	-3.81584	-0.446	-2.46594
H	-3.87143	-2.2238	-2.50768
H	-5.367	-1.26637	-2.68416

Table S7: Cartesian coordinates of **TPD6.**

Atom	X-axis	Y-axis	Z-axis
C	-3.96811	0.954459	0.138204
C	-3.39604	-0.29669	0.043655
C	-5.43152	-1.35684	-0.6428
C	-6.11848	-0.09181	-0.17604
C	-5.39115	1.035416	0.127122
C	-7.50354	0.160018	-0.08069
C	-7.81685	1.462258	0.281173
S	-8.94205	-0.77512	-0.33914
C	-9.20018	1.729316	0.342816
C	-9.94669	0.625397	0.030463
H	-9.641	2.689528	0.582718
C	-1.9947	-0.23979	0.126139
C	-1.52115	1.052459	0.29988
S	-0.73054	-1.4276	0.087797
C	-0.12169	1.107882	0.393255
C	0.452975	-0.14909	0.289858
S	-6.38172	2.413532	0.53597
S	-2.79318	2.234788	0.330339
C	1.863081	-0.22152	0.2108
C	4.270768	-0.63508	-0.1244
C	3.972695	0.679074	0.277272
S	2.81829	-1.59304	-0.2847
S	5.403862	1.625005	0.412142
C	2.607948	0.916745	0.47238

C	5.614622	-0.89189	-0.33025
H	6.050193	-1.82846	-0.6521
C	6.401113	0.232336	-0.07384
O	-4.09684	-1.43957	-0.04812
O	0.57057	2.247075	0.483885
C	1.943087	2.169523	0.99641
C	7.780748	0.472473	-0.16015
H	8.048084	1.498954	0.085468
C	8.855532	-0.34105	-0.44331
C	8.886779	-1.78985	-0.65449
C	10.2321	0.122425	-0.53283
C	11.11156	-1.05775	-0.31783
C	12.43485	-1.24684	0.098171
C	12.95979	-2.55485	0.013769
C	12.16769	-3.62662	-0.38258
C	10.8172	-3.4561	-0.63336
C	10.31721	-2.17869	-0.58664
H	10.16233	-4.29394	-0.84703
O	7.974603	-2.57512	-0.81365
C	10.61444	1.337846	-1.04001
C	-6.16929	-2.57359	-0.12642
C	-6.80106	-3.48277	-0.9631
C	-6.27093	-2.75523	1.25473
C	-7.53033	-4.54203	-0.4323
H	-6.74594	-3.37229	-2.04125
C	-6.99362	-3.80933	1.776594
H	-5.78021	-2.05032	1.920849
C	-7.64267	-4.72131	0.939836
H	-8.02351	-5.24048	-1.10363
H	-7.06701	-3.93292	2.854676
C	2.642063	3.396132	0.452123
C	3.328928	4.290848	1.261574
C	2.656561	3.59864	-0.92915
C	4.03464	5.349773	0.701257
H	3.336455	4.168213	2.339714
C	3.356277	4.654031	-1.4805
H	2.117585	2.909346	-1.57397
C	4.070821	5.542924	-0.67393
H	4.572168	6.036368	1.350383
H	3.361299	4.793195	-2.55892
C	9.669166	2.326573	-1.43734
N	8.928886	3.147412	-1.77405
C	11.94461	1.67496	-1.40704

N	12.98823	1.995368	-1.78325
C	4.871205	6.649791	-1.28483
H	4.328622	7.129713	-2.10498
H	5.127611	7.414199	-0.54692
H	5.809028	6.264295	-1.70177
C	-8.42644	-5.85601	1.521081
H	-8.92794	-6.43766	0.743606
H	-7.77691	-6.53505	2.084441
H	-9.18835	-5.49248	2.218784
C	-11.4016	0.517096	0.054296
C	-12.0935	-0.25435	-0.93364
C	-12.1259	1.153347	1.02588
C	-13.5547	1.079008	1.095226
H	-11.6042	1.712224	1.798041
C	-14.2227	0.299418	0.130734
C	-14.3401	1.706809	2.068269
H	-13.8539	2.311431	2.82843
C	-15.7197	1.558187	2.061555
H	-16.3116	2.053943	2.824607
C	-11.7483	-0.9913	-2.06552
H	-10.745	-1.12136	-2.44302
C	-12.9201	-1.51271	-2.62546
H	-12.9951	-2.13012	-3.50897
C	-13.9965	-1.0958	-1.85002
C	-15.4056	-1.2606	-1.88058
H	-15.8376	-1.86362	-2.672
C	-16.1875	-0.67074	-0.94102
H	-17.266	-0.79453	-0.9662
C	-15.6214	0.138881	0.103615
C	-16.3584	0.789451	1.097957
H	-17.4396	0.685603	1.104252
N	-13.4726	-0.32612	-0.82486
H	12.60935	-4.61359	-0.44131
C	13.18345	-0.16727	0.856713
C	14.37586	-2.93217	0.398309
F	14.19315	0.375331	0.191966
F	12.3623	0.821452	1.217959
F	13.6776	-0.67752	1.989718
F	14.4512	-3.28686	1.683234
F	15.25968	-1.96349	0.189015
F	14.78712	-3.98528	-0.3144
C	1.855415	2.110145	2.510202
H	1.3602	3.002076	2.904841

H	1.284347	1.227003	2.811064
H	2.854563	2.027985	2.949234
C	-5.2601	-1.3168	-2.15052
H	-4.66107	-0.44404	-2.42651
H	-4.75423	-2.21876	-2.50741
H	-6.23107	-1.22691	-2.64735

Table S8: Calculated energies (E) and energy gap (ΔE) of HOMO-1/LUMO+1 and HOMO-2/LUMO+2 for **TPR** and **TPD1-TPD6**.

Compounds	HOMO-1	LUMO+1	ΔE	HOMO-2	LUMO+2	ΔE
**TPR**	-6.474	-3.294	3.18	-6.931	-2.815	4.116
**TPD1**	-5.617	-2.607	3.01	-6.289	-2.155	4.134
**TPD2**	-5.657	-2.999	2.658	-6.324	-2.257	4.067
**TPD3**	-5.671	-3.271	2.4	-6.381	-2.868	3.513
**TPD4**	-5.623	-2.723	2.9	-6.381	-2.186	4.195
**TPD5**	-5.613	-2.751	2.862	-6.298	-2.181	4.117
**TPD6**	-5.612	-2.867	2.745	-6.300	-2.289	4.011

Table S9: Wavelength, excitation energy and oscillator strength of **TPR** at M06/6-311G(d,p) level in the solvent phase.

NO	DFT λ (nm)	$E(eV)$	f_{os}	MO contributions
1	749.602	1.654	3.120	H→L (95%), H-1→L+1 (3%)
2	602.099	2.059	0.001	H→L+1 (97%),
3	509.364	2.434	0.138	H-1→L (15%), H→L+2 (75%), H-1→L+3 (5%)
4	508.695	2.437	0.008	H-1→L (79%), H→L+2 (15%),
5	487.877	2.541	0.001	H→L+3 (88%), H-1→L+2 (6%)
6	454.471	2.728	0.373	H-2→L (17%), H-1→L+1 (75%), H→L (4%)

MO=molecular orbital, H=HOMO, L=LUMO, f_{os}= oscillator strength, λ (nm)= wavelength

Table S10: Wavelength, excitation energy and oscillator strength of **TPD1** at M06/6-311G(d,p) level in the solvent phase.

NO	DFT λ (nm)	$E(eV)$	f_{os}	MO contributions
1	719.709	1.723	2.023	H-1→L (19%), H→L (77%),
2	609.229	2.035	0.180	H-1→L (76%), H→L (21%),
3	523.383	2.369	0.341	H-1→L+1 (21%), H→L+1 (69%), H-2→L+1 (3%), H→L+2 (3%)
4	485.622	2.553	0.123	H-2→L (87%), H-3→L (3%), H-1→L (2%), H→L+2 (4%)
5	467.865	2.650	0.054	H-1→L+1 (36%), H→L+2 (43%), H-2→L (3%), H-2→L+1 (2%), H-1→L+3 (3%), H→L+1 (5%), H→L+3 (3%)
6	452.927	2.737	0.196	H-1→L+1 (27%), H-1→L+2 (10%), H→L+1 (20%), H→L+2 (38%), H-2→L (2%)

MO=molecular orbital, H=HOMO, L=LUMO, f_{os}= oscillator strength, wavelength= λ (nm)

Table S11: Wavelength, excitation energy and oscillator strength of **TPD2** at M06/6-311G(d,p) level in the solvent phase.

NO	DFT λ (*nm*)	*E*(*eV*)	f_{os}	MO contributions
1	801.294	1.547	1.556	H-1→L (20%), H→L (77%),
2	691.105	1.794	0.274	H-1→L (76%), H→L (21%),
3	601.369	2.062	0.630	H-1→L+1 (25%), H→L+1 (68%),
4	529.011	2.344	0.078	H-1→L+1 (62%), H→L+1 (27%), H-2→L (6%)
5	526.092	2.357	0.051	H-2→L (84%), H-3→L (3%), H-1→L+1 (6%)
6	466.790	2.656	0.315	H→L+2 (56%), H→L+3 (26%), H-1→L+2 (2%), H-1→L+4 (2%), H→L+1 (2%), H→L+4 (4%)

MO=molecular orbital, H=HOMO, L=LUMO, f_{os}= oscillator strength, wavelength= λ (*nm*)

Table S12: Wavelength, excitation energy and oscillator strength of **TPD3** at M06/6-311G(d,p) level in the solvent phase.

NO	DFT λ (*nm*)	*E*(*eV*)	f_{os}	MO contributions
1	833.619	1.487	1.579	H-1→L (15%), H→L (82%),
2	717.958	1.727	0.432	H-1→L (81%), H→L (17%),
3	644.945	1.922	0.065	H-1→L+1 (23%), H→L+1 (73%), H-2→L+1 (3%)
4	568.266	2.182	0.047	H-1→L+1 (67%), H→L+1 (26%), H-2→L+1 (2%)
5	558.815	2.219	0.574	H-2→L (11%), H-1→L+2 (14%), H→L+2 (60%), H-1→L+1 (4%), H→L+4 (2%)
6	536.264	2.312	0.042	H-2→L (78%), H→L+2 (14%), H-3→L (4%)

MO=molecular orbital, H=HOMO, L=LUMO, f_{os}= oscillator strength, wavelength= λ (*nm*)

Table S13: Wavelength, excitation energy and oscillator strength of **TPD4** at M06/6-311G(d,p) level in the solvent phase.

NO	DFT λ (*nm*)	*E*(*eV*)	f_{os}	MO contributions
1	743.177	1.668	1.980	H-1→L (18%), H→L (78%),
2	632.218	1.961	0.257	H-1→L (78%), H→L (20%),
3	541.203	2.291	0.281	H-1→L+1 (21%), H→L+1 (70%), H-2→L+1 (3%), H→L+2 (2%)
4	499.091	2.484	0.247	H-2→L (86%), H-3→L (4%), H-1→L (3%), H→L+2 (4%)
5	477.910	2.594	0.002	H-1→L+1 (62%), H→L+1 (18%), H→L+2 (11%), H-2→L+1 (3%)
6	463.544	2.675	0.228	H→L+2 (70%), H-2→L (4%), H-1→L+1 (6%), H-1→L+2 (4%), H→L+1 (9%), H→L+3 (3%)

MO=molecular orbital, H=HOMO, L=LUMO, f_{os}= oscillator strength, wavelength= λ (*nm*)

Table S14: Wavelength, excitation energy and oscillator strength of **TPD5** at M06/6-311G(d,p) level in the solvent phase.

NO	DFT λ (*nm*)	$E(eV)$	f_{os}	MO contributions
1	758.731	1.634	1.940	H-1→L (17%), H→L (79%),
2	644.007	1.925	0.213	H-1→L (79%), H→L (19%),
3	551.752	2.247	0.342	H-1→L+1 (21%), H→L+1 (71%), H-2→L+1 (3%)
4	506.906	2.446	0.143	H-2→L (88%), H-3→L (3%), H-1→L (2%), H→L+2 (2%)
5	483.595	2.564	0.003	H-1→L+1 (66%), H→L+1 (20%), H-2→L+1 (2%), H→L+2 (7%)
6	463.926	2.673	0.295	H→L+2 (76%), H-2→L (3%), H-1→L+1 (3%), H-1→L+2 (3%), H→L+1 (6%), H→L+3 (3%)

MO=molecular orbital, H=HOMO, L=LUMO, f_{os}= oscillator strength, wavelength= λ (*nm*)

Table S15: Wavelength, excitation energy and oscillator strength of **TPD6** at M06/6-311G(d,p) level in the solvent phase.

NO	DFT λ (*nm*)	$E(eV)$	f_{os}	MO contributions
1	768.465	1.613	1.901	H-1→L (16%), H→L (80%),
2	650.051	1.907	0.208	H-1→L (80%), H→L (18%),
3	576.456	2.151	0.182	H-1→L+1 (22%), H→L+1 (72%), H-2→L+1 (4%)
4	511.570	2.424	0.253	H-2→L (83%), H-3→L (3%), H-1→L (3%), H→L+2 (6%)
5	500.360	2.478	0.007	H-1→L+1 (70%), H→L+1 (24%), H-2→L+1 (2%)
6	479.982	2.583	0.354	H→L+2 (76%), H-2→L (8%), H-1→L+2 (4%), H→L+3 (5%)

MO=molecular orbital, H=HOMO, L=LUMO, f_{os}= oscillator strength, wavelength= λ (*nm*)

Table S16: Wavelength, excitation energy and oscillator strength of **TPR** at M06/6-311G(d,p) level in the gaseous phase.

NO	DFT λ (*nm*)	$E(eV)$	f_{os}	MO contributions
1	692.688	1.790	2.852	H→L (96%), H-1→L+1 (3%)
2	567.070	2.186	0.002	H→L+1 (97%),
3	490.095	2.530	0.056	H-1→L (69%), H→L+2 (14%), H→L+3 (12%),
4	488.588	2.538	0.076	H-1→L (16%), H→L+2 (76%), H-1→L+3 (5%)
5	468.661	2.646	0.003	H-1→L (12%), H→L+3 (78%), H-1→L+2 (5%)
6	440.379	2.815	0.325	H-2→L (11%), H-1→L+1 (78%), H→L (3%), H→L+4 (6%)

MO=molecular orbital, H=HOMO, L=LUMO, f_{os}= oscillator strength, wavelength= λ (*nm*)

Table S17: Wavelength, excitation energy and oscillator strength of **TPD1** at M06/6-311G(d,p) level in the gaseous phase.

**NO**	**DFT** λ (*nm*)	*E*(*eV*)	f_{os}	**MO contributions**
1	666.223	1.861	1.785	H-1→L (11%), H→L (86%),
2	581.676	2.132	0.252	H-1→L (86%), H→L (11%),
3	503.939	2.460	0.347	H-1→L+1 (17%), H→L+1 (73%), H-2→L+1 (3%), H→L+2 (3%)
4	470.832	2.633	0.161	H-2→L (68%), H→L+2 (19%), H-1→L+1 (3%)
5	456.025	2.719	0.008	H-2→L (14%), H-1→L+1 (30%), H→L+2 (28%), H-1→L+2 (9%), H-1→L+3 (3%), H→L+1 (4%), H→L+3 (3%)
6	438.169	2.830	0.130	H-2→L (10%), H-1→L+1 (28%), H-1→L+2 (10%), H→L+1 (13%), H→L+2 (37%),

MO=molecular orbital, H=HOMO, L=LUMO, f_{os}= oscillator strength, wavelength= λ (*nm*)

Table S18: Wavelength, excitation energy and oscillator strength of **TPD2** at M06/6-311G(d,p) level in the gaseous phase.

**NO**	**DFT** λ (*nm*)	*E* (*eV*)	f_{os}	**MO contributions**
1	737.167	1.682	1.202	H-1→L (11%), H→L (88%),
2	657.985	1.884	0.461	H-1→L (87%), H→L (11%),
3	572.517	2.166	0.580	H-1→L+1 (24%), H→L+1 (70%),
4	513.158	2.416	0.056	H-1→L+1 (69%), H→L+1 (25%),
5	504.678	2.457	0.158	H-2→L (87%), H-3→L (3%), H-1→L+2 (2%), H→L+2 (4%)
6	458.963	2.701	0.162	H→L+2 (76%), H-2→L (5%), H-1→L+4 (3%), H→L+1 (2%), H→L+3 (2%), H→L+4 (5%)

MO=molecular orbital, H=HOMO, L=LUMO, f_{os}= oscillator strength, wavelength= λ (*nm*)

Table S19: Wavelength, excitation energy and oscillator strength of **TPD3** at M06/6-311G(d,p) level in the gaseous phase.

**NO**	**DFT** λ (*nm*)	*E*(*eV*)	f_{OS}	**MO contributions**
1	765.335	1.620	1.090	H→L (93%), H-1→L (6%)
2	679.031	1.826	0.792	H-1→L (92%), H→L (6%)
3	607.438	2.041	0.046	H-1→L+1 (24%), H→L+1 (72%), H-2→L+1 (2%)
4	544.268	2.278	0.013	H-1→L+1 (69%), H→L+1 (27%), H-2→L+1 (2%)
5	524.934	2.362	0.676	H-2→L (39%), H-1→L+2 (10%), H→L+2 (38%), H-3→L (2%), H→L+3 (6%)
6	501.757	2.471	0.003	H-2→L (50%), H→L+2 (42%),

MO=molecular orbital, H=HOMO, L=LUMO, f_{os}= oscillator strength, wavelength= λ (*nm*)

Table S20: Wavelength, excitation energy and oscillator strength of **TPD4** at M06/6-311G(d,p) level in the gaseous phase.

NO	DFT λ (*nm*)	*E*(*eV*)	f_{os}	MO contributions
1	691.490	1.793	1.631	H-1→L (10%), H→L (88%),
2	610.579	2.031	0.427	H-1→L (87%), H→L (11%),
3	519.480	2.387	0.280	H-1→L+1 (21%), H→L+1 (70%), H-2→L+1 (3%), H→L+2 (2%)
4	483.992	2.562	0.324	H-2→L (73%), H→L+2 (15%), H-3→L (2%), H-1→L+2 (3%)
5	465.459	2.664	0.006	H-1→L+1 (55%), H→L+1 (15%), H→L+2 (13%), H-2→L (4%), H-1→L+2 (5%)
6	450.377	2.753	0.083	H-2→L (13%), H-1→L+1 (12%), H→L+1 (10%), H→L+2 (56%), H→L+3 (3%)

MO=molecular orbital, H=HOMO, L=LUMO, f_{os}= oscillator strength, wavelength= λ (*nm*)

Table S21: Wavelength, excitation energy and oscillator strength of **TPD5** at M06/6-311G(d,p) level in the gaseous phase.

NO	DFT λ (*nm*)	*E*(*eV*)	f_{os}	MO contributions
1	702.579	1.765	1.575	H-1→L (10%), H→L (88%),
2	622.254	1.993	0.395	H-1→L (87%), H→L (11%),
3	529.214	2.343	0.350	H-1→L+1 (22%), H→L+1 (70%), H-2→L+1 (3%)
4	490.075	2.530	0.203	H-2→L (80%), H-3→L (2%), H-1→L+2 (2%), H→L+2 (9%)
5	472.357	2.625	0.006	H-1→L+1 (62%), H→L+1 (19%), H-1→L+2 (3%), H→L+2 (8%)
6	452.118	2.742	0.148	H-2→L (10%), H→L+2 (67%), H-1→L+1 (6%), H-1→L+3 (2%), H→L+1 (7%), H→L+3 (4%)

MO=molecular orbital, H=HOMO, L=LUMO, f_{os}= oscillator strength, wavelength= λ (*nm*)

Table S22: Wavelength, excitation energy and oscillator strength of **TPD6** at M06/6-311G(d,p) level in the gaseous phase.

NO	DFT λ (*nm*)	*E*(*eV*)	f_{os}	MO contributions
1	711.939	1.742	1.544	H-1→L (10%), H→L (88%),
2	629.106	1.971	0.395	H-1→L (88%), H→L (10%),
3	554.095	2.238	0.136	H-1→L+1 (23%), H→L+1 (71%), H-2→L+1 (3%)
4	497.469	2.492	0.416	H-2→L (66%), H→L+2 (21%), H-3→L (2%), H-1→L+2 (3%)
5	488.531	2.538	0.020	H-1→L+1 (67%), H→L+1 (26%), H-2→L+1 (2%)
6	466.949	2.655	0.133	H-2→L (23%), H→L+2 (64%), H→L+3 (4%)

MO=molecular orbital, H=HOMO, L=LUMO, f_{os}= oscillator strength, wavelength= λ (*nm*)

Table S23: Natural bond orbitals analysis for **TPR** with its representative values.

**Donor(*i*)**	**Type**	**Acceptor(*j*)**	***Type***	***E*(2) [*kcal/mol*]**	***E*(*j*)-*E*(*i*) [*a.u*]**	***F*(*i,j*) [*a.u*]**
C9-C10	π	C30-C32	π*	28.62	0.31	0.084
C25-C27	π	C44-C46	π*	28.42	0.31	0.084
C30-C32	π	C33-C59	π*	26.32	0.3	0.08
C44-C46	π	C47-O57	π*	23.14	0.31	0.077
C50-C51	π	C49-C54	π*	21.2	0.32	0.075
C12-C13	π	C1-C2	π*	19.23	0.31	0.072
C1-C2	π	C4-C5	π*	18.3	0.32	0.071
C19-C24	π	C15-C16	π*	11.75	0.3	0.055
C48-C60	π	C49-C54	π*	8.56	0.33	0.05
C47-O57	π	C49-C54	π*	4.2	0.42	0.042
C34-O58	π	C30-C32	π*	3.7	0.43	0.038
C30-C32	π	C30-C32	π*	2.47	0.31	0.025
C4-C5	π	C4-C5	π*	1.01	0.31	0.016
C87-N88	π	C85-N86	π*	0.78	0.47	0.017
C85-N86	π	C87-N88	π*	0.77	0.47	0.017
C59-C63	σ	C63-N64	σ*	8.21	1.61	0.103
C63-N64	σ	C59-C63	σ*	8.11	1.57	0.102
C60-C85	σ	C85-N86	σ*	8.1	1.61	0.103
C15-C16	σ	C13-S18	σ*	7.02	0.93	0.072
C20-C21	σ	C24-C31	σ*	6.3	1.15	0.076
C4-C5	σ	C1-C5	σ*	6.02	1.27	0.078
S23-C27	σ	C21-C24	σ*	5.7	1.2	0.074
C65-C66	σ	C65-C67	σ*	5	1.3	0.072
C38-C39	σ	C37-C38	σ*	4.06	1.32	0.065
C80-C82	σ	C77-C80	σ*	4.01	1.3	0.065
C65-C67	σ	C3-C65	σ*	3.08	1.13	0.053
C49-C50	σ	C50-C51	σ*	2.97	1.29	0.055
C5-S17	σ	C1-C2	σ*	2.3	1.23	0.048
C38-H42	σ	C39-F97	σ*	1.5	0.84	0.032
C65-C67	σ	C67-H71	σ*	1.3	1.12	0.034
C76-C78	σ	C78-H83	σ*	1.25	1.13	0.034
C5-S17	σ	C4-C5	σ*	1.1	1.25	0.033
C61-N62	σ	C33-C59	σ*	0.52	1.65	0.026
C16-C19	σ	C19-S22	σ*	0.51	0.94	0.02
C1-C2	σ	C3-O28	σ*	0.5	0.98	0.02
O28	LP(2)	C1-C2	π*	34.46	0.35	0.106
S22	LP(2)	C19-C24	π*	26.94	0.27	0.078
S18	LP(2)	C12-C13	π*	25.56	0.25	0.076
S17	LP(2)	C6-C7	π*	22.73	0.25	0.071
S8	LP(2)	C9-C10	π*	19.56	0.27	0.066
S23	LP(2)	C25-C27	π*	19.24	0.27	0.066
O58	LP(2)	C34-C36	σ*	20.82	0.75	0.113
N62	LP(1)	C59-C61	σ*	12.68	1.04	0.103
F97	LP(2)	C39-C40	σ*	7.6	0.98	0.077
O28	LP(1)	C3-C4	σ*	3.36	0.98	0.051
O57	LP(1)	C47-C54	σ*	1.9	1.18	0.043
S14	LP(1)	C16-C19	σ*	0.51	1.2	0.022

Table S24: Natural bond orbitals analysis for **TPD1** with its representative values.

**Donor(*i*)**	**Type**	**Acceptor(*j*)**	**Type**	***E*(2) [*kcal/mol*]**	***E*(*j*)-*E*(*i*) [*a.u*]**	***F*(*i,j*) [*a.u*]**
C25-C27	π	C31-C33	π*	29.04	0.31	0.085
C31-C33	π	C35-C45	π*	26.65	0.3	0.08
C59-C63	π	C56-C57	π*	24.96	0.29	0.076
C31-C33	π	C34-O44	π*	23.16	0.31	0.077
C36-C37	π	C40-C41	π*	21.5	0.31	0.073
C86-C98	π	C81-C84	π*	20.12	0.29	0.07
C56-C57	π	C59-C63	π*	18.9	0.31	0.069
C6-C7	π	C4-C5	π*	17.09	0.32	0.068
C9-C10	π	C6-C7	π*	15.16	0.29	0.065
C15-C16	π	C12-C13	π*	13.08	0.29	0.06
C35-C45	π	C31-C33	π*	7.81	0.33	0.046
C34-O44	π	C40-C41	π*	4.1	0.44	0.041
C68-N69	π	C66-N67	π*	0.81	0.47	0.017
C66-N67	π	C68-N69	π*	0.79	0.47	0.017
C15-C16	π	C15-C16	π*	0.61	0.31	0.013
C45 - C68	σ	C68 - N69	σ*	8.18	1.61	0.103
C25 - C27	σ	C20 - S22	σ*	8.09	0.92	0.077
C68 - N69	σ	C45 - C68	σ*	8.08	1.57	0.101
C15 - C16	σ	C13 - S18	σ*	7.04	0.93	0.073
C88 - C90	σ	C78 - C79	σ*	6.77	1.21	0.081
C79 - C88	σ	C83 - N 100	σ*	5.01	1.15	0.068
C48 - H52	σ	C46 - C47	σ*	4.8	1.11	0.065
C80 - C81	σ	C78 - C80	σ*	4	1.29	0.064
C92 - N 100	σ	C83 - N 100	σ*	3.59	1.25	0.06
C81 - C84	σ	C84 - C86	σ*	3.4	1.3	0.06
C38 - C39	σ	C39 - C40	σ*	3.1	1.3	0.057
C88 - C90	σ	C79 - C88	σ*	2.99	1.25	0.055
C 3 - C46	σ	C 3 - C 4	σ*	2	1.06	0.041
C21 - S23	σ	C21 - C24	σ*	1.85	1.26	0.043
C31 - C33	σ	C35 - C36	σ*	1.4	1.22	0.037
C83 - N 100	σ	C79 - C88	σ*	1.08	1.41	0.035
C57 - H60	σ	C57 - C59	σ*	0.89	1.12	0.028
C12 - S14	σ	C15 - C16	σ*	0.5	1.23	0.022
C15 - C16	σ	O29 - C30	σ*	0.5	0.98	0.02
C20 - C21	σ	C21 - S23	σ*	0.5	0.94	0.019
N 100	LP (1)	C83 - C97	π*	43.95	0.31	0.106
O28	LP (2)	C 1 - C 2	π*	28.63	0.36	0.097
S 8	LP (2)	C 6 - C 7	π*	25.09	0.26	0.075
S17	LP (2)	C 6 - C 7	π*	24.64	0.26	0.074
S18	LP (2)	C 1 - C 2	π*	22.34	0.27	0.071
S23	LP (2)	C25 - C27	π*	19.17	0.27	0.065
O44	LP (2)	C34 - C41	σ*	20.31	0.76	0.112
N69	LP (1)	C45 - C68	σ*	12.65	1.04	0.103
O29	LP (2)	C30 - C 107	σ*	5.29	0.74	0.058
S14	LP (1)	C15 - C16	σ*	2.79	1.21	0.052
S18	LP (1)	C13 - C15	σ*	0.7	1.21	0.026
S18	LP (1)	C 1 - C 5	σ*	0.51	1.2	0.022

Table S25: Natural bond orbitals analysis for **TPD2** with its representative values.

**Donor(*i*)**	**Type**	**Acceptor(*j*)**	**Type**	***E*(2) [*kcal/mol*]**	***E*(*j*)-*E*(*i*) [*a.u*]**	***F*(*i,j*) [*a.u*]**
C25-C27	π	C31-C33	π*	31.48	0.3	0.087
C38-C39	π	N102-O103	π*	28.54	0.16	0.064
C31-C33	π	C35-C45	π*	28.42	0.29	0.082
C51-C53	π	C46-C48	π*	24.11	0.3	0.075
C47-C49	π	C51-C53	π*	22.72	0.31	0.075
C56-C57	π	C58-C61	π*	21.02	0.31	0.072
C12-C13	π	C15-C16	π*	20.69	0.31	0.075
C86-C98	π	C81-C84	π*	20.15	0.29	0.07
C6-C7	π	C4-C5	π*	17.31	0.31	0.069
C19-C24	π	C15-C16	π*	11.74	0.3	0.055
C34-O44	π	C31-C33	π*	3.6	0.43	0.038
C19-C24	π	C19-C24	π*	1.37	0.31	0.019
C68-N69	π	C66-N67	π*	0.8	0.47	0.017
C66-N67	π	C68-N69	π*	0.78	0.47	0.017
C15-C16	π	C15-C16	π*	0.75	0.31	0.014
C45-C68	σ	C68-N69	σ*	8.22	1.61	0.103
C25-C27	σ	C20-S22	σ*	8.18	0.92	0.077
C68-N69	σ	C45-C68	σ*	8.14	1.57	0.102
C1-C2	σ	C12-S14	σ*	7.06	0.93	0.073
C12-C13	σ	C15-O29	σ*	6.33	1.12	0.075
C35-C45	σ	C45-C68	σ*	6.02	1.27	0.078
C25-H26	σ	S23-C27	σ*	5.8	0.72	0.058
C20-C21	σ	C20-C25	σ*	5	1.31	0.072
C98-H99	σ	C84-C86	σ*	3.97	1.11	0.059
C46-C47	σ	C48-H52	σ*	2.8	1.12	0.05
C3-C4	σ	C3-C105	σ*	1.9	1.05	0.04
C38-C39	σ	C38-H101	σ*	1.37	1.16	0.036
C48-C51	σ	C51-H55	σ*	1.2	1.12	0.033
C3-C4	σ	C105-H108	σ*	1.16	1.05	0.031
N102-O103	σ	C39-N102	σ*	1.1	1.4	0.036
C36-C37	σ	C33-C35	σ*	1.01	1.24	0.032
C1-C2	σ	C3-O28	σ*	0.6	0.99	0.022
C66-N67	σ	C35-C45	σ*	0.52	1.64	0.026
C38-C39	σ	C39-N102	σ*	0.51	1.02	0.021
C12-S14	σ	C15-C16	σ*	0.5	1.23	0.022
N100	LP(1)	C83-C97	π*	43.96	0.31	0.106
S23	LP(2)	C20-C21	π*	28.93	0.24	0.079
S8	LP(2)	C6-C7	π*	25.2	0.26	0.075
S18	LP(2)	C12-C13	π*	24.58	0.25	0.074
S14	LP(2)	C15-C16	π*	20.36	0.27	0.068
S23	LP(2)	C25-C27	π*	18.52	0.27	0.064
O44	LP(2)	C34-C41	σ*	20.95	0.75	0.113
N67	LP(1)	C45-C66	σ*	12.68	1.04	0.103
O103	LP(1)	C39-N102	σ*	4.16	1.09	0.062
S18	LP(1)	C12-C13	σ*	2.7	1.2	0.051
O28	LP(2)	C2-C12	σ*	1.6	0.89	0.035
S18	LP(1)	C1-C5	σ*	0.51	1.2	0.022

Table S26: Natural bond orbitals analysis for **TPD3** with its representative values.

**Donor(*i*)**	**Type**	**Acceptor(*j*)**	**Type**	***E*(2) [*kcal/mol*]**	***E*(*j*)-*E*(*i*) [*a.u*]**	***F*(*i,j*) [*a.u*]**
C25-C27	π	C31-C33	π*	31.66	0.3	0.088
C20-C21	π	C25-C27	π*	25.76	0.3	0.08
C31-C33	π	C34-O43	π*	25.24	0.3	0.08
C47-C50	π	C48-C52	π*	23.11	0.31	0.075
C80-C83	π	C82-C96	π*	22.79	0.29	0.073
C12-C13	π	C15-C16	π*	21.12	0.31	0.075
C60-C62	π	C56-C58	π*	20.99	0.3	0.071
C82-C96	π	C80-C83	π*	19.71	0.3	0.069
C6-C7	π	C4-C5	π*	17.52	0.32	0.069
C1-C2	π	C4-C5	π*	15.3	0.32	0.065
C4-C5	π	C1-C2	π*	13	0.3	0.059
N104-O106	π	C38-C39	π*	2.25	0.52	0.033
C67-N68	π	C65-N66	π*	0.79	0.47	0.017
C65-N66	π	C67-N68	π*	0.73	0.48	0.017
C15-C16	π	C15-C16	π*	0.7	0.3	0.014
S23-C27	σ	C21-C24	σ*	5.66	1.2	0.074
C19-C24	σ	S14-C16	σ*	5.64	0.92	0.065
C107-H110	σ	C3-O28	σ*	5.64	0.76	0.059
C89-C91	σ	C82-N99	σ*	4.9	1.15	0.067
C79-C80	σ	C77-C79	σ*	4	1.29	0.064
C69-H71	σ	C60-C62	σ*	3.42	1.1	0.055
C36-C41	σ	C35-C36	σ*	3.1	1.16	0.054
C91-N99	σ	C77-C78	σ*	3.02	1.33	0.057
C3-C107	σ	C45-C47	σ*	2.9	1.23	0.053
C77-C78	σ	C91-N99	σ*	2.7	1.13	0.049
C3-C45	σ	C2-O28	σ*	2.52	1.02	0.045
C20-C25	σ	C25-H26	σ*	2.3	1.16	0.046
C78-N99	σ	C77-C78	σ*	1.98	1.33	0.046
C12-S14	σ	C1-C2	σ*	1.76	1.24	0.042
C30-C55	σ	C111-H113	σ*	1.46	1.03	0.035
C45-C47	σ	C47-H51	σ*	1.3	1.12	0.034
N104-O106	σ	C38-C39	σ*	0.8	1.67	0.033
C12-C13	σ	C13-S18	σ*	0.51	0.93	0.019
C12-S14	σ	C15-C16	σ*	0.51	1.23	0.022
C13-S18	σ	C1-C2	σ*	0.51	1.24	0.022
N99	LP(1)	C82-C96	π*	43.98	0.31	0.106
O28	LP(2)	C1-C2	π*	31.25	0.36	0.101
S14	LP(2)	C12-C13	π*	25.8	0.25	0.076
S18	LP(2)	C1-C2	π*	22.53	0.27	0.072
O105	LP(2)	N104-O106	π*	2.52	0.2	0.023
O106	LP(2)	C38-C39	π*	0.85	0.32	0.016
O43	LP(2)	C34-C41	σ*	21.65	0.73	0.114
O102	LP(2)	C37-N101	σ*	15.83	0.57	0.085
N68	LP(1)	C44-C67	σ*	12.87	1.04	0.103
S18	LP(1)	C1-C2	σ*	2.87	1.22	0.053
O43	LP(1)	C25-H26	σ*	1.5	1.14	0.037
S14	LP(1)	C16-C19	σ*	0.5	1.21	0.022

Table S27: Natural bond orbitals analysis for **TPD4** with its representative values.

**Donor(*i*)**	**Type**	**Acceptor(*j*)**	**Type**	***E*(2) [*kcal/mol*]**	***E*(*j*)-*E*(*i*) [*a.u*]**	***F*(*i,j*) [*a.u*]**
C25-C27	π	C31-C33	π*	29.86	0.31	0.086
C31-C33	π	C35-C45	π*	27.26	0.3	0.081
C20-C21	π	C25-C27	π*	24.94	0.31	0.08
C40-C41	π	C38-C39	π*	22.88	0.28	0.072
C46-C47	π	C48-C51	π*	20.97	0.31	0.072
C83-C97	π	C81-C84	π*	19.71	0.3	0.069
C81-C84	π	C78-C80	π*	17.21	0.3	0.066
C1-C2	π	C12-C13	π*	15.11	0.29	0.064
C4-C5	π	C1-C2	π*	12.71	0.3	0.059
C35-C45	π	C36-C37	π*	9.1	0.33	0.05
C35-C45	π	C31-C33	π*	7.87	0.33	0.046
C19-C24	π	C19-C24	π*	1.3	0.3	0.018
C68-N69	π	C66-N67	π*	0.84	0.47	0.018
C66-N67	π	C68-N69	π*	0.82	0.47	0.018
C15-C16	π	C15-C16	π*	0.61	0.31	0.013
C45-C68	σ	C68-N69	σ*	8.15	1.61	0.103
C45-C66	σ	C66-N67	σ*	8.13	1.61	0.103
C68-N69	σ	C45-C68	σ*	8.06	1.57	0.101
C1-C2	σ	C12-S14	σ*	7.02	0.94	0.073
C88-C90	σ	C78-C79	σ*	6.76	1.21	0.081
C35-C45	σ	C45-C68	σ*	6.06	1.27	0.078
C4-C5	σ	C4-C6	σ*	5.98	1.28	0.078
C21-C24	σ	C19-C24	σ*	5	1.29	0.072
C10-C78	σ	C78-C79	σ*	4.1	1.23	0.064
C45-C66	σ	C68-N69	σ*	3.8	1.61	0.07
C83-N100	σ	C79-N100	σ*	3.4	1.27	0.059
C88-C90	σ	C79-C88	σ*	2.99	1.25	0.055
C34-C41	σ	C34-O44	σ*	2.01	1.25	0.045
C78-C80	σ	C80-H82	σ*	1.4	1.14	0.036
C25-C27	σ	C31-H32	σ*	1.3	1.13	0.034
C31-H32	σ	C31-C33	σ*	1.13	1.12	0.032
C5-S17	σ	C1-C5	σ*	0.8	1.21	0.028
C107-H109	σ	C3-O28	σ*	0.56	0.76	0.019
C66-N67	σ	C35-C45	σ*	0.52	1.65	0.026
C12-S14	σ	C15-C16	σ*	0.51	1.23	0.022
N100	LP(1)	C83-C97	π*	43.97	0.31	0.106
O29	LP(2)	C15-C16	π*	31.93	0.36	0.103
S22	LP(2)	C19-C24	π*	27.03	0.27	0.078
S18	LP(2)	C12-C13	π*	24.52	0.25	0.074
S14	LP(2)	C15-C16	π*	20.6	0.27	0.068
S23	LP(2)	C25-C27	π*	18.56	0.27	0.065
O44	LP(2)	C34-C41	σ*	20.69	0.75	0.113
O29	LP(1)	C15-C16	σ*	7.75	1.12	0.083
O44	LP(1)	C33-C34	σ*	3.16	1.17	0.055
S17	LP(1)	C4-C5	σ*	2.97	1.24	0.054
S8	LP(1)	C4-C6	σ*	0.91	1.21	0.03
Cl102	LP(2)	C37-C38	σ*	0.5	0.91	0.019

Table S28: Natural bond orbitals analysis for **TPD5** with its representative values.

**Donor(*i*)**	**Type**	**Acceptor(*j*)**	**Type**	***E*(2) [*kcal/mol*]**	***E*(*j*)-*E*(*i*) [*a.u*]**	***F*(*i,j*) [*a.u*]**
C25-C27	π	C31-C33	π*	30.59	0.31	0.087
C31-C33	π	C35-C45	π*	27.53	0.3	0.081
C20-C21	π	C25-C27	π*	25.24	0.3	0.08
C48-C51	π	C49-C53	π*	23	0.31	0.075
C40-C41	π	C36-C37	π*	22.01	0.3	0.074
C38-C39	π	C40-C41	π*	21.53	0.32	0.074
C15-C16	π	C19-C24	π*	20.13	0.31	0.074
C38-C39	π	C36-C37	π*	19.12	0.31	0.069
C79-C88	π	C78-C80	π*	18.66	0.31	0.068
C81-C84	π	C78-C80	π*	17.21	0.3	0.066
C20-C21	π	C19-C24	π*	16.4	0.31	0.064
C9-C10	π	C6-C7	π*	15.13	0.29	0.065
C68-N69	π	C66-N67	π*	0.77	0.47	0.017
C66-N67	π	C68-N69	π*	0.76	0.47	0.017
C15-C16	π	C15-C16	π*	0.72	0.31	0.014
C45-C68	σ	C68-N69	σ*	8.23	1.61	0.103
C25-C27	σ	C20-S22	σ*	8.17	0.92	0.077
C68-N69	σ	C45-C68	σ*	8.13	1.57	0.102
C4-C5	σ	C6-S8	σ*	7.72	0.94	0.076
C31-C33	σ	C27-C31	σ*	6.4	1.31	0.082
C35-C45	σ	C45-C68	σ*	6.03	1.27	0.078
C4-C5	σ	C4-C6	σ*	5.97	1.28	0.078
C79-C88	σ	C83-N100	σ*	5.03	1.15	0.068
C5-S17	σ	C3-C4	σ*	4.8	1.09	0.065
C80-C81	σ	C78-C80	σ*	4	1.29	0.064
C92-N100	σ	C83-N100	σ*	3.6	1.25	0.06
C2-C12	σ	C13-C15	σ*	3.1	1.27	0.056
C79-N100	σ	C92-N100	σ*	2.9	1.23	0.054
C10-C78	σ	C7-C9	σ*	2	1.24	0.045
C40-H43	σ	C40-C41	σ*	1.5	1.13	0.037
C58-H62	σ	C56-C58	σ*	1	1.1	0.03
C53-C74	σ	C74-H77	σ*	0.7	1.03	0.024
C66-N67	σ	C35-C45	σ*	0.52	1.64	0.026
C13-S18	σ	C1-C2	σ*	0.51	1.24	0.023
C40-H43	σ	C39-C102	σ*	0.5	0.91	0.019
N100	LP(1)	C83-C97	π*	44.02	0.31	0.106
O28	LP(2)	C1-C2	π*	31.17	0.36	0.101
S14	LP(2)	C12-C13	π*	25.71	0.25	0.076
S8	LP(2)	C9-C10	π*	23.54	0.28	0.073
S18	LP(2)	C1-C2	π*	22.43	0.27	0.071
F104	LP(2)	C38-C39	π*	0.79	0.48	0.019
O44	LP(2)	C34-C41	σ*	20.78	0.75	0.113
N69	LP(1)	C45-C68	σ*	12.67	1.04	0.103
F103	LP(2)	C39-C102	σ*	6.21	0.83	0.064
O28	LP(1)	C3-C4	σ*	3.3	0.98	0.051
S14	LP(1)	C2-C12	σ*	0.7	1.21	0.026
F105	LP(1)	C39-C102	σ*	0.53	1.48	0.025

Table S29: Natural bond orbitals analysis for **TPD6** with its representative values.

**Donor(*i*)**	***Type***	**Acceptor(*j*)**	***Type***	***E*(2) [*kcal/mol*]**	***E*(*j*)-*E*(*i*) [*a.u*]**	***F*(*i,j*) [*a.u*]**
C25-C27	π	C31-C33	π*	30.07	0.31	0.086
C58-C62	π	C55-C56	π*	24.86	0.29	0.076
C20-C21	π	C25-C27	π*	24.8	0.31	0.079
C40-C41	π	C38-C39	π*	22.57	0.29	0.074
C55-C56	π	C57-C60	π*	21.02	0.31	0.072
C85-C97	π	C80-C83	π*	20.1	0.29	0.07
C78-C87	π	C77-C79	π*	18.65	0.31	0.068
C80-C83	π	C77-C79	π*	17.22	0.3	0.066
C1-C2	π	C4-C5	π*	15.3	0.32	0.065
C36-C37	π	C35-C44	π*	11.04	0.31	0.053
C77-C79	π	C9-C10	π*	9.7	0.3	0.05
C31-C33	π	C31-C33	π*	2.26	0.31	0.024
C67-N68	π	C65-N66	π*	0.82	0.47	0.018
C65-N66	π	C67-N68	π*	0.73	0.48	0.017
C15-C16	π	C15-C16	π*	0.71	0.31	0.014
C44-C67	σ	C67-N68	σ*	8.4	1.62	0.105
C67-N68	σ	C44-C67	σ*	8.37	1.57	0.103
C25-C27	σ	C20-S22	σ*	8.17	0.92	0.078
C15-C16	σ	C13-S18	σ*	7.04	0.93	0.073
C12-C13	σ	C15-O29	σ*	6.3	1.12	0.075
C4-C5	σ	C4-C6	σ*	5.98	1.28	0.078
C55-C57	σ	C55-C56	σ*	4.9	1.29	0.071
C47-C50	σ	C50-C52	σ*	4.2	1.3	0.066
C79-C80	σ	C77-C79	σ*	4	1.29	0.064
C20-C25	σ	C21-C24	σ*	3.1	1.3	0.057
C91-N99	σ	C77-C78	σ*	3.02	1.33	0.057
C78-N99	σ	C91-N99	σ*	2.9	1.23	0.053
C45-C46	σ	C47-H51	σ*	2.4	1.13	0.047
C15-O29	σ	S14-C16	σ*	1.8	1.15	0.041
C87-C89	σ	C89-H90	σ*	1.4	1.1	0.035
C47-H51	σ	C45-C47	σ*	1	1.1	0.03
C113-H115	σ	C3-O28	σ*	0.57	0.76	0.019
C67-N68	σ	C35-C44	σ*	0.54	1.63	0.027
C101-F105	σ	C101-F103	σ*	0.52	1.34	0.024
C102-F107	σ	C102-F106	σ*	0.51	1.35	0.024
N99	LP(1)	C82-C96	π*	44.02	0.31	0.106
O28	LP(2)	C1-C2	π*	31.06	0.36	0.101
S8	LP(2)	C6-C7	π*	25.52	0.26	0.075
S18	LP(2)	C1-C2	π*	22.44	0.27	0.071
S23	LP(2)	C25-C27	π*	19.18	0.27	0.065
F105	LP(2)	C36-C37	π*	0.63	0.47	0.017
O43	LP(2)	C34-C41	σ*	21.31	0.74	0.113
N66	LP(1)	C44-C65	σ*	12.7	1.04	0.103
F107	LP(2)	C38-C102	σ*	7.12	0.81	0.068
O29	LP(2)	C30-C109	σ*	5.22	0.74	0.057
S17	LP(1)	C4-C5	σ*	2.98	1.24	0.054
F104	LP(1)	C37-C101	σ*	0.53	1.45	0.025

Table S30: Dipole polarizability and major contributing tensor (*esu*) of the studied compounds (**TPR** and **TPD1-TPD6**).

System	μ_x	μ_y	μ_z	μ_{total}
**TPR**	0.996	0.963	1.593	2.111
**TPD1**	10.070	-3.326	-1.592	10.724
**TPD2**	15.961	1.888	-0.005	16.073
**TPD3**	23.105	4.540	5.467	24.173
**TPD4**	14.040	2.870	3.432	14.735
**TPD5**	-14.327	-1.376	2.009	14.532
**TPD6**	10.803	-0.239	-0.262	10.809

Table S31: Linear polarizability $\langle\alpha\rangle$ and major contributing tensor (*esu*) of the studied compounds (**TPR** and **TPD1-TPD6**).

System	α_{xx} ($\times 10^{-22}$)	α_{yy} ($\times 10^{-22}$)	α_{zz} ($\times 10^{-22}$)	$\langle\alpha\rangle$ ($\times 10^{-22}$)
**TPR**	6.23	1.80	0.89	2.97
**TPD1**	4.99	1.70	1.03	2.57
**TPD2**	5.50	1.70	1.10	2.76
**TPD3**	5.84	0.01	1.10	2.89
**TPD4**	5.23	1.82	0.99	2.69
**TPD5**	5.30	1.71	1.07	2.69
**TPD6**	5.33	1.69	1.12	2.72

Table S32: The computed first hyperpolarizability (β_{tot}) and major contributing tensors (*esu*) of the studied compounds (**TPR** and **TPD1-TPD6**).

First Hyperpolarizability	TPR	TPD1	TPD2	TPD3	TPD4	TPD5	TPD6
β_{xxx} ($\times 10^{-27}$)	0.274	3.56	5.31	7.18	4.46	-4.67	-4.99
β_{xxy} ($\times 10^{-28}$)	0.329	1.92	1.71	-3.46	-1.08	0.343	1.95
β_{xyy} ($\times 10^{-29}$)	0.112	0.221	2.23	4.04	-2.10	0.541	-1.99
β_{yyy} ($\times 10^{-30}$)	4.59	1.34	27.6	-7.21	17.3	8.04	11.3
β_{xxz} ($\times 10^{-29}$)	7.00	2.14	-8.99	11.7	5.25	11.5	25.4
β_{yyz} ($\times 10^{-30}$)	2.60	5.68	12.2	-6.23	2.51	-3.80	-2.16
β_{xzz} ($\times 10^{-30}$)	5.59	-0.63	5.75	17.0	1.47	-6.94	-16.6
β_{yzz} ($\times 10^{-30}$)	0.036	0.87	0.23	-2.51	1.69	-2.88	-2.05
β_{zzz} ($\times 10^{-30}$)	2.26	2.56	3.67	-0.16	1.89	-3.46	-3.21
β_{tot} ($\times 10^{-27}$)	0.29	3.56	5.34	7.24	4.44	4.68	5.04

Table S33: Second hyperpolarizability $<\gamma>$ and major contributing tensor (*esu*) of the studied compounds (**TPR** and **TPD1-TPD6**).

System	γ_x ($\times 10^{-32}$)	γ_y ($\times 10^{-34}$)	γ_z ($\times 10^{-35}$)	$<\gamma>$ ($\times 10^{-32}$)
**TPR**	6.29	1.94	5.18	6.31
**TPD1**	4.39	1.39	5.50	4.41
**TPD2**	6.95	3.32	8.54	7.00
**TPD3**	9.96	4.03	16.8	10.0
**TPD4**	5.40	1.29	3.75	5.40
**TPD5**	5.66	0.75	3.09	5.67
**TPD6**	6.23	2.13	9.98	6.25

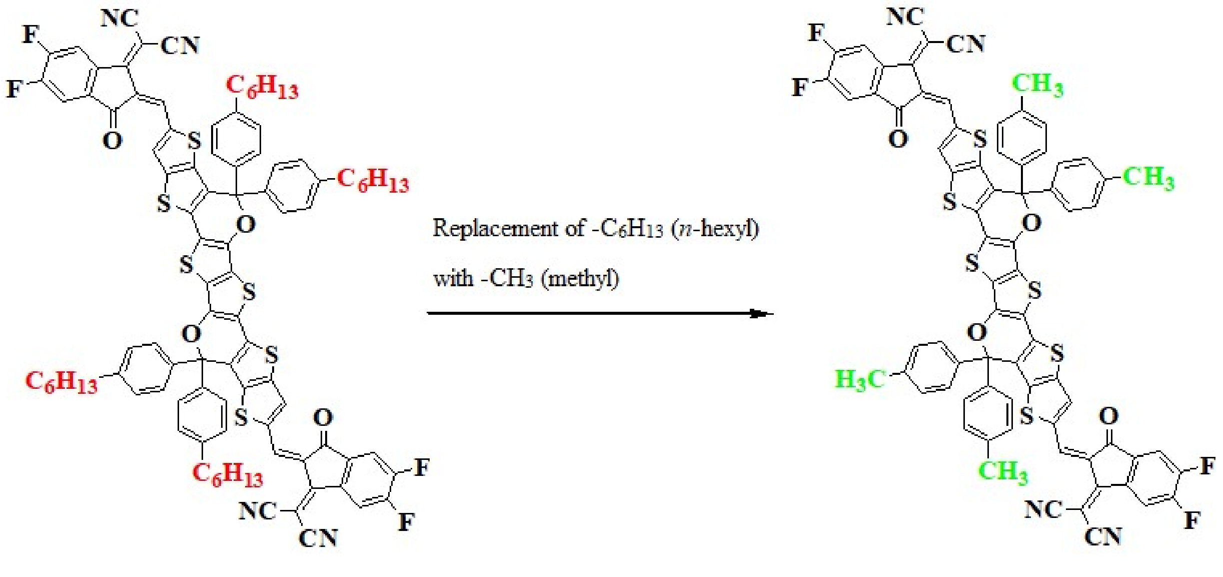

COi8DFIC (Parent) **TPR (Reference)**

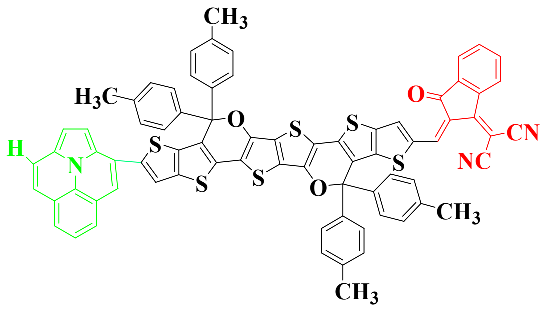

TPD1

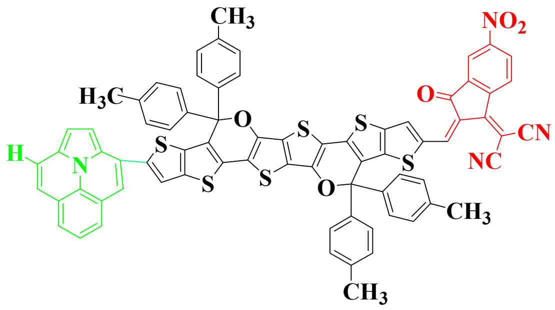

TPD2

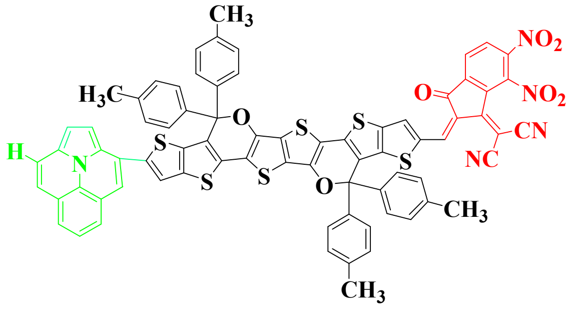

TPD3

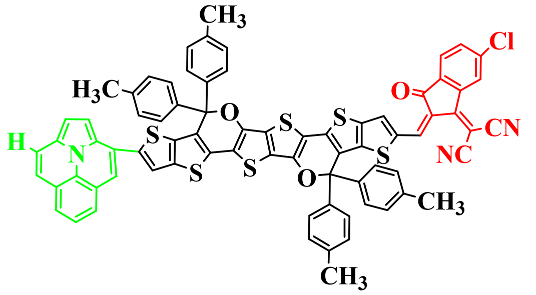

TPD4

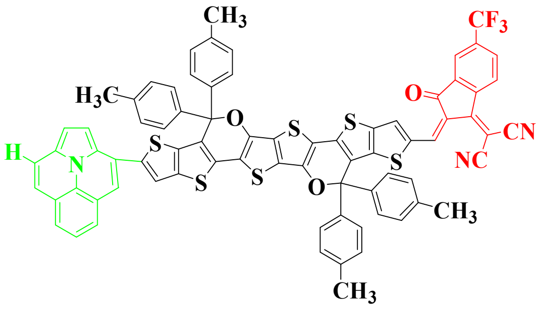

TPD5

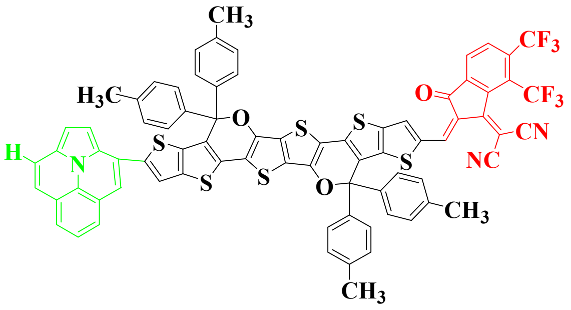

TPD6

Figure S1: Chemdraw structures of **TPR** and **TPD1-TPD6**.

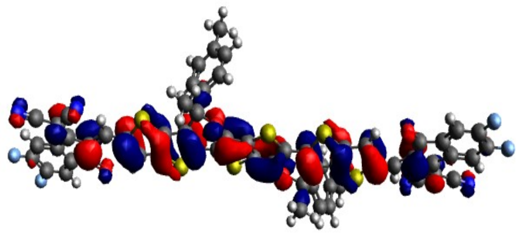

HOMO -1

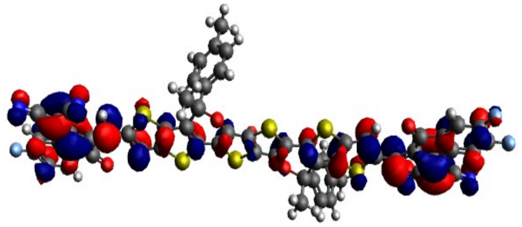

LUMO +1

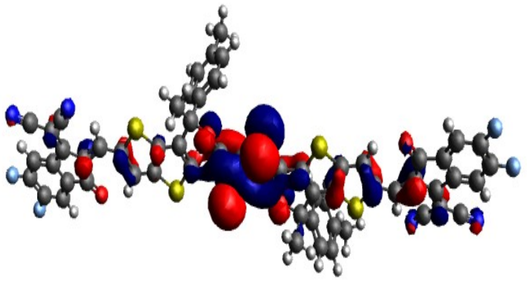

HOMO -2

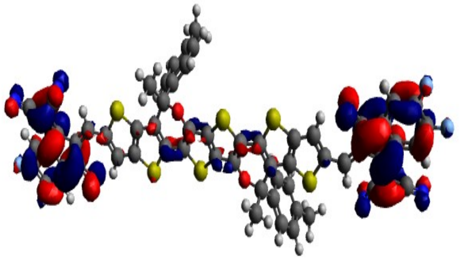

LUMO +2

TPR

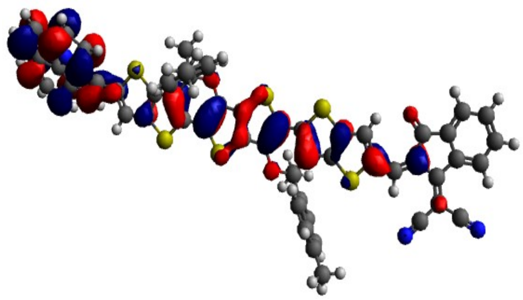

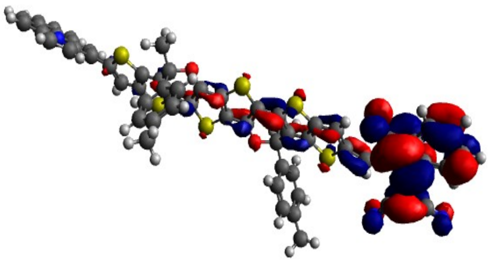

HOMO -1 **LUMO +1**

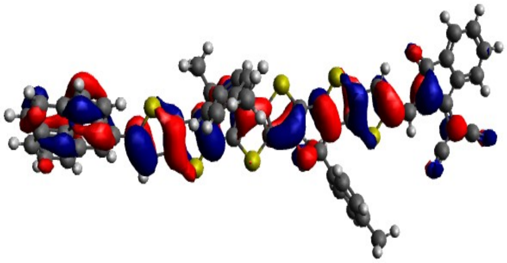

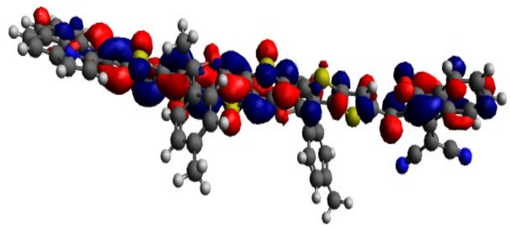

HOMO -2 **LUMO +2**

TPD1

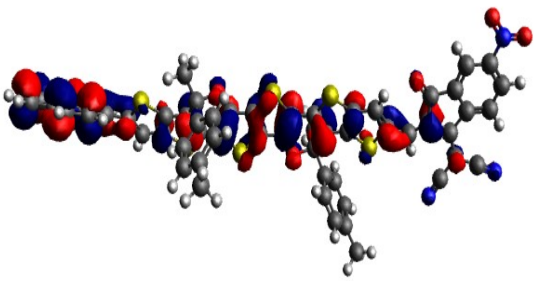

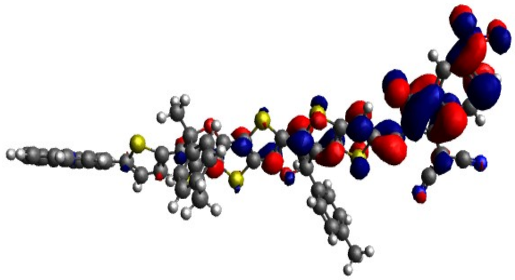

HOMO -1 **LUMO +1**

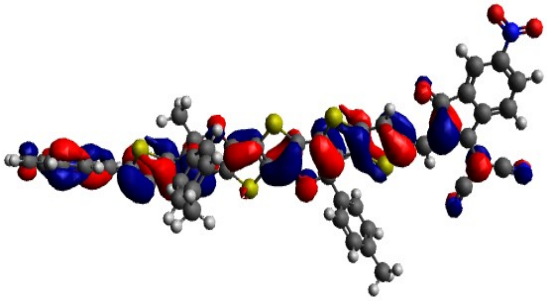

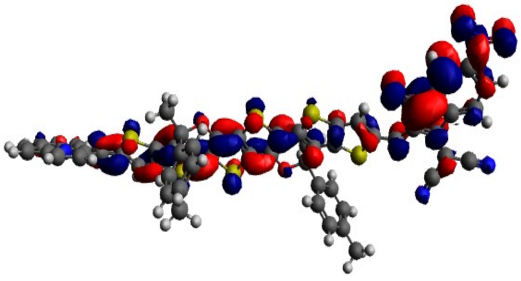

HOMO -2 **LUMO +2**

TPD2

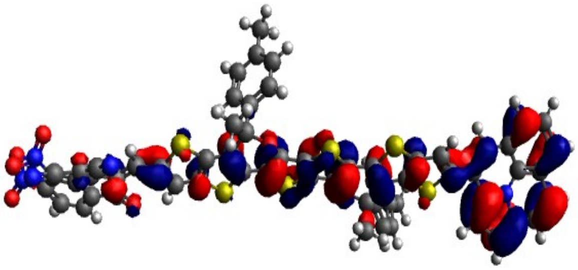

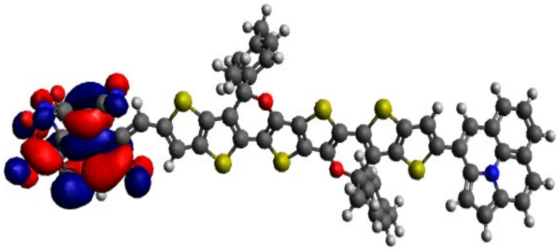

HOMO -1 **LUMO +1**

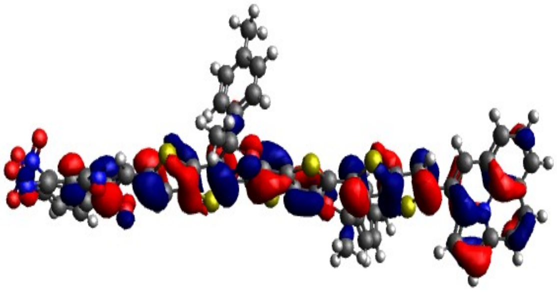

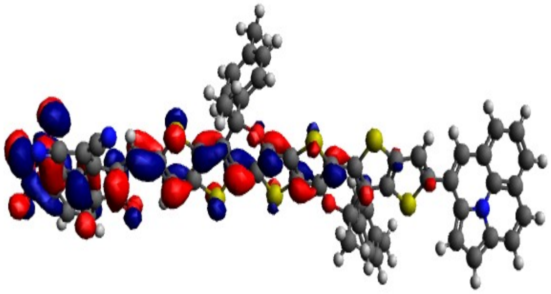

HOMO -2 **LUMO +2**

TPD3

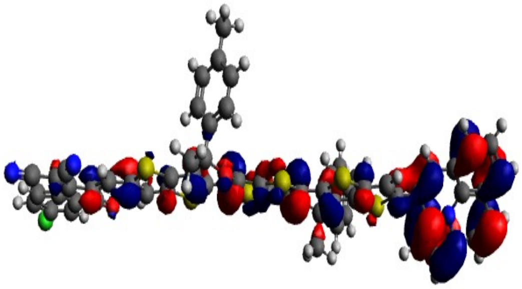

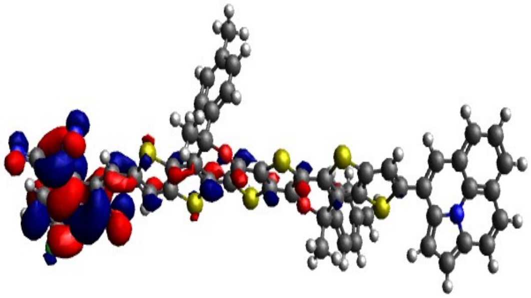

HOMO -1 **LUMO +1**

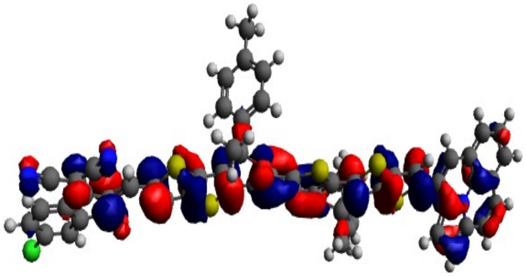

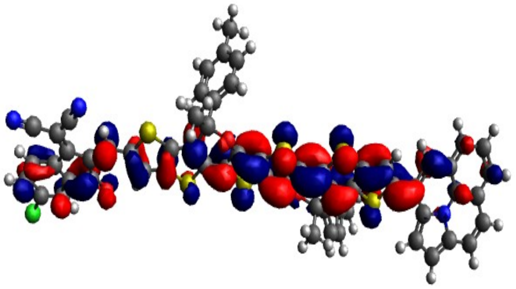

HOMO -2 **LUMO +2**

TPD4

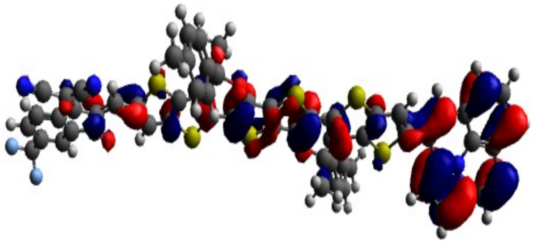

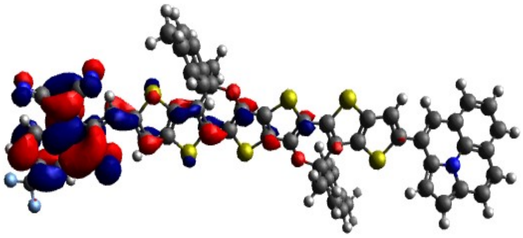

HOMO -1 **LUMO +1**

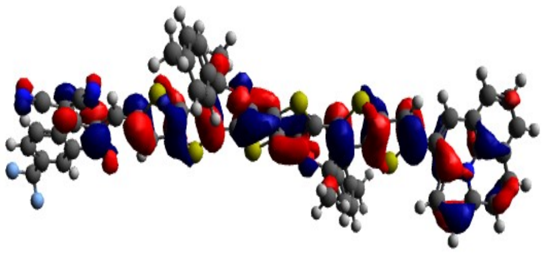 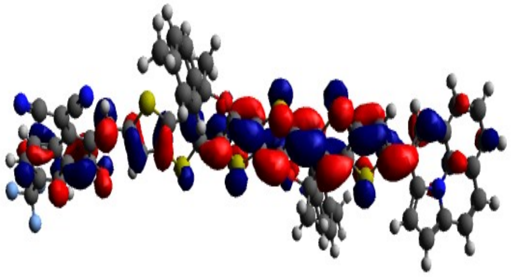

HOMO -2 **LUMO +2**

TPD5

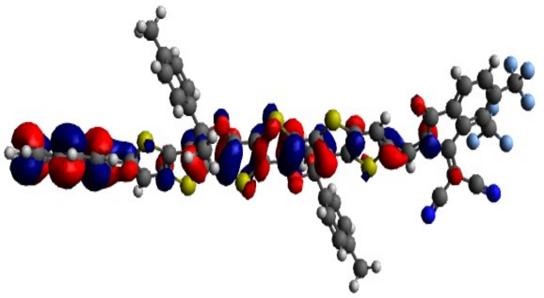 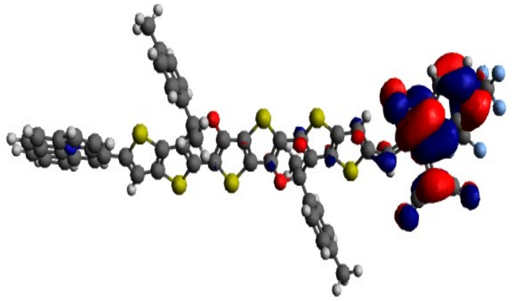

HOMO -1 **LUMO +1**

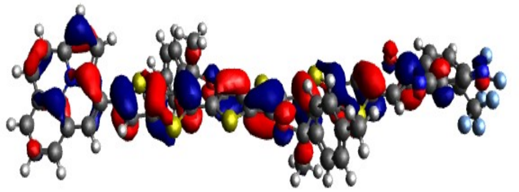 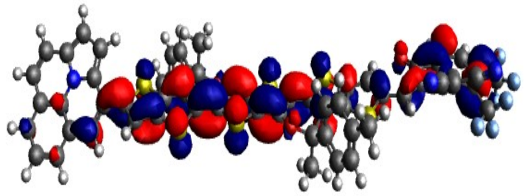

HOMO -2 **LUMO +2**

TPD6

Figure S2: Graphical representation of HOMO-1/LUMO+1 and HOMO-2/LUMO+2 of **TPR** and **TPD1-TPD6**.

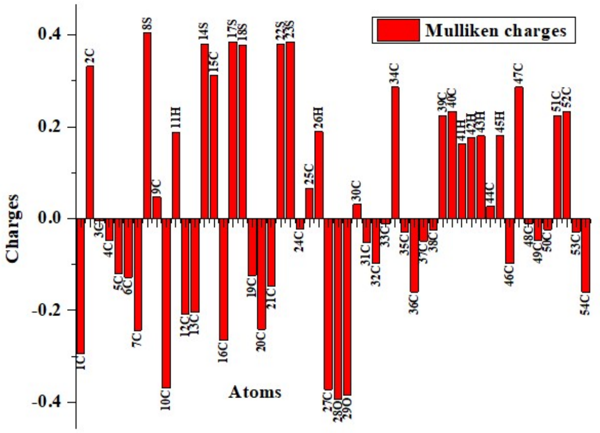

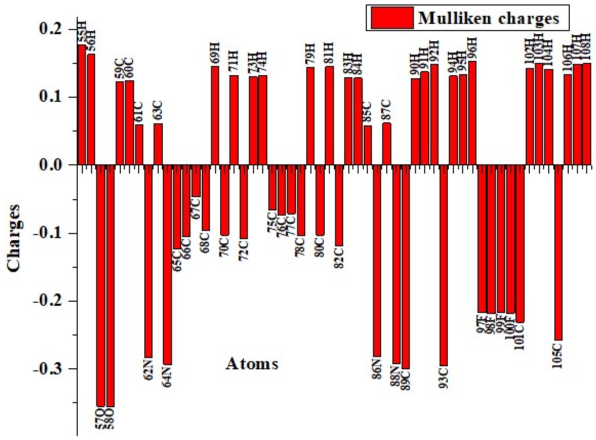

1st half

2nd half

TPR

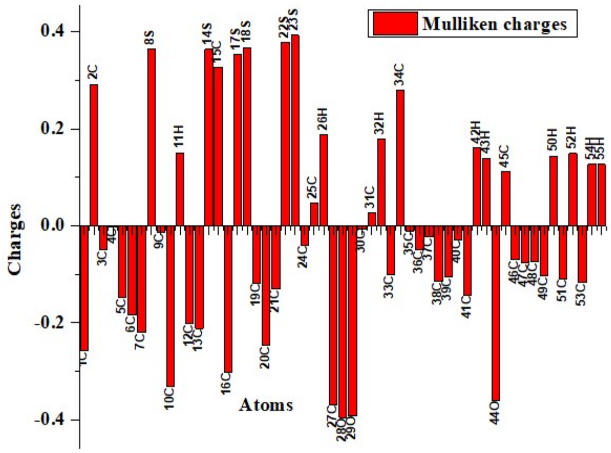

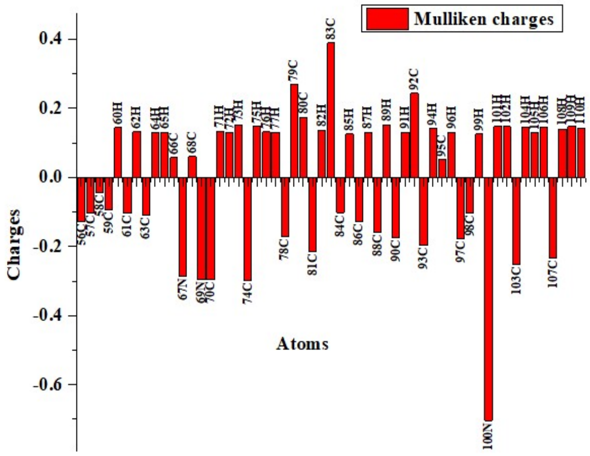

1st half

2nd half

TPD1

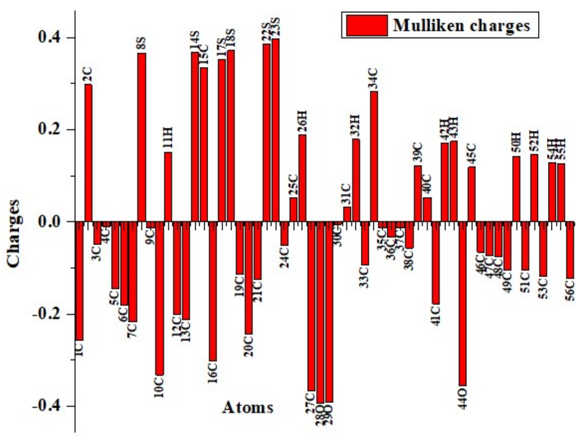

1st half

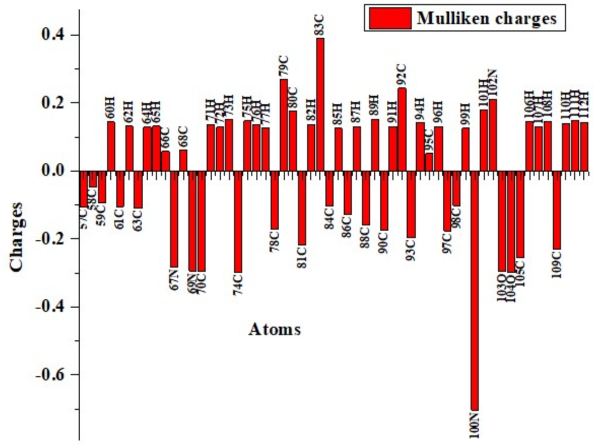

2nd half

TPD2

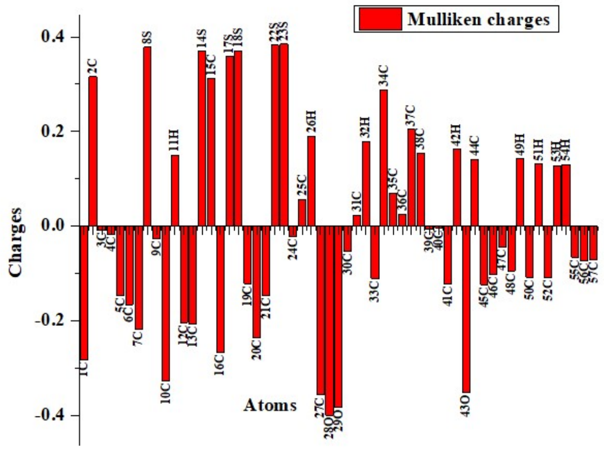

1st half

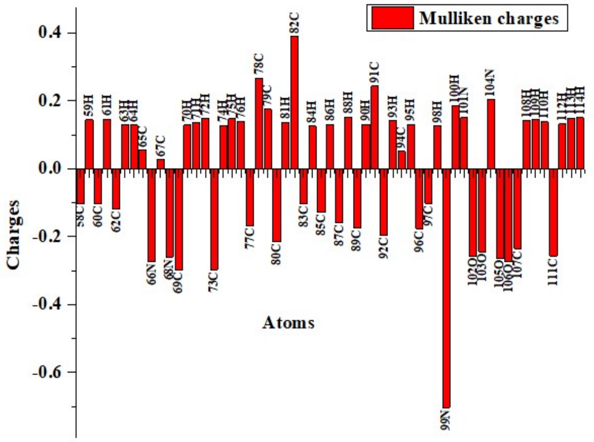

2nd half

TPD3

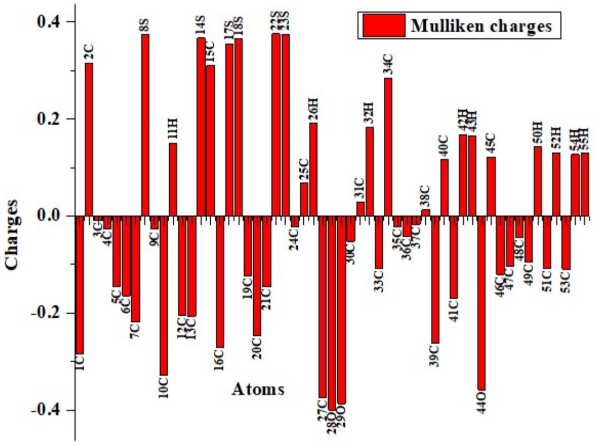

1st half

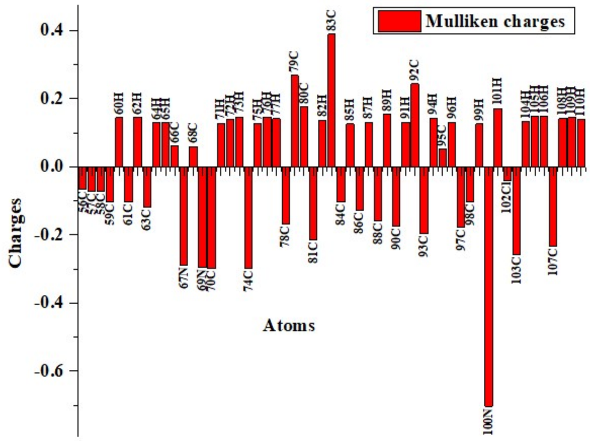

2nd half

TPD4

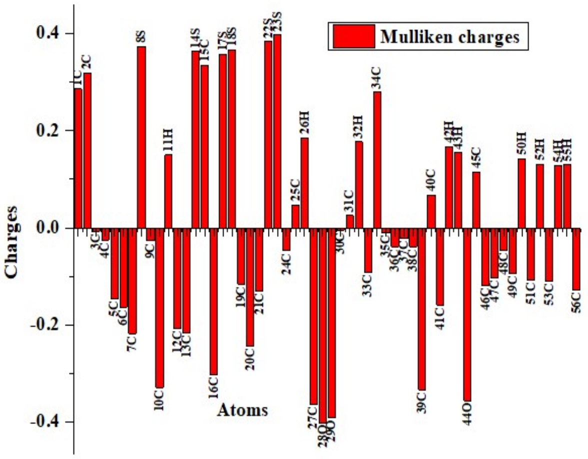

1st half

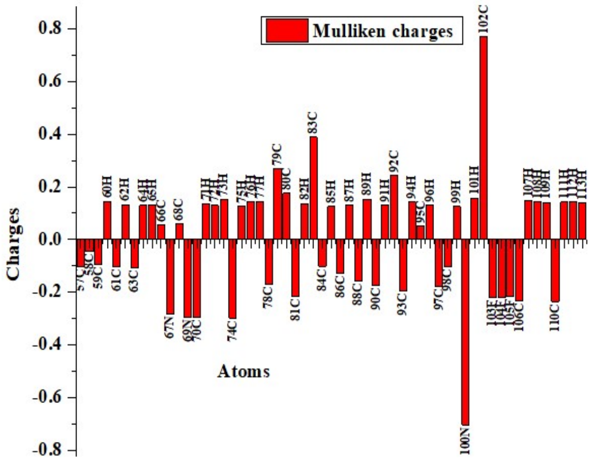

2nd half

TPD5

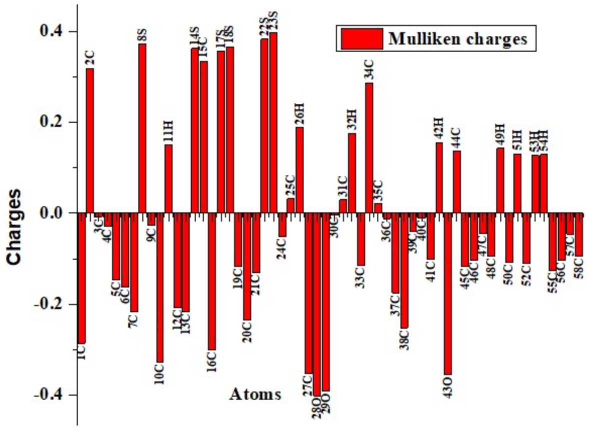

1st half

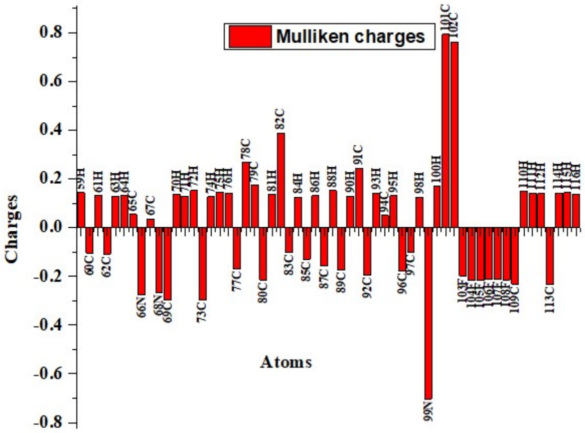

2nd half

TPD6

Figure S3: Graphical representation of natural population analysis of investigated compounds (**TPR** and **TPD1-TPD6**).
